# Comparative genomic analysis of *Staphylococcus lugdunensis* shows a closed pan-genome and multiple barriers to horizontal gene transfer

**DOI:** 10.1186/s12864-018-4978-1

**Published:** 2018-08-20

**Authors:** Xavier Argemi, Dorota Matelska, Krzysztof Ginalski, Philippe Riegel, Yves Hansmann, Jochen Bloom, Martine Pestel-Caron, Sandrine Dahyot, Jérémie Lebeurre, Gilles Prévost

**Affiliations:** 1 Service des Maladies Infectieuses et Tropicales, Hôpitaux Universitaires, Nouvel Hôpital Civil, 1 Place de l’Hôpital, 67000 Strasbourg, France; 2 Université de Strasbourg, CHRU Strasbourg, Fédération de Médecine Translationnelle de Strasbourg, EA 7290, Virulence Bactérienne Précoce, F-67000 Strasbourg, France; 3 Laboratory of Bioinformatics and Systems Biology, Centre of New Technologies, University of Warsaw, Zwirki i Wigury 93, 02-089 Warsaw, Poland; 4 Bioinformatics & Systems Biology, Justus-Liebig-University Gießen, 35392 Gießen, Germany; 5 Normandie Univ, UNIROUEN, GRAM EA2656, Rouen University Hospital, F-76000 Rouen, France

**Keywords:** *Staphylococcus lugdunensis*, Comparative genomics, Pan genome, Core genome, Toxin/antitoxin, Restriction-modification, CRISPR

## Abstract

**Background:**

Coagulase negative staphylococci (CoNS) are commensal bacteria on human skin. *Staphylococcus lugdunensis* is a unique CoNS which produces various virulence factors and may, like *S. aureus*, cause severe infections, particularly in hospital settings. Unlike other staphylococci, it remains highly susceptible to antimicrobials, and genome-based phylogenetic studies have evidenced a highly conserved genome that distinguishes it from all other staphylococci.

**Results:**

We demonstrate that *S. lugdunensis* possesses a closed pan-genome with a very limited number of new genes, in contrast to other staphylococci that have an open pan-genome. Whole-genome nucleotide and amino acid identity levels are also higher than in other staphylococci. We identified numerous genetic barriers to horizontal gene transfer that might explain this result. The *S. lugdunensis* genome has multiple operons encoding for restriction-modification, CRISPR/Cas and toxin/antitoxin systems. We also identified a new PIN-like domain-associated protein that might belong to a larger operon, comprising a metalloprotease, that could function as a new toxin/antitoxin or detoxification system.

**Conclusion:**

We show that *S. lugdunensis* has a unique genome profile within staphylococci, with a closed pan-genome and several systems to prevent horizontal gene transfer. Its virulence in clinical settings does not rely on its ability to acquire and exchange antibiotic resistance genes or other virulence factors as shown for other staphylococci.

**Electronic supplementary material:**

The online version of this article (10.1186/s12864-018-4978-1) contains supplementary material, which is available to authorized users.

## Background

*Staphylococcus lugdunensis* is a commensal bacterium found on human skin that has been reported as a cause of severe infections in hospital and community settings [1]. Its clinical virulence clearly distinguishes this coagulase-negative staphylococcus (CoNS) from others in the genus. It appears closest to *S. aureus* in terms of clinical significance and virulence; infection rates may reach 40% when, typically, hospital microbiology laboratories consider infection rates of 25% or less for other CoNS [2]. In vitro studies have revealed the existence of several putative virulence factors, such as haemolysins, adhesion proteins, and one protease that might constitute the cornerstone of *S. lugdunensis* virulence [3, 4]. Recent genomic studies have demonstrated some general characteristics of this particular CoNS. Its genome is closer to that of *S. aureus* than other CoNS, possessing several mobile genetic elements (MGEs) such as plasmids and prophages, which have been described at a genetic level in seven strains, although these do not seem to support the virulence profile of this bacterium [5, 6]. In contrast, MGEs in the form of plasmids, phages, phage-related chromosomal islands (PRCIs, including *S. aureus* pathogenicity islands SaPIs), transposons, staphylococcal cassette chromosomes (SCCs), integrative and conjugative elements, accounting for up to 25% of the genome of *S. aureus*, are also widespread in other CoNS, and play a crucial role in the modulation of their virulence [7]. Surprisingly, *S. lugdunensis*, in contrast to all other staphylococci, displays a highly conserved antibiotic sensitivity profile, and methicillin resistance is extremely rare even in hospital settings, notwithstanding the few SCC *mec*-bearing strains that have been described [3, 8, 9]. Prophages, plasmids, and SaPIs usually bear *S. aureus* pore-forming toxins and superantigen enterotoxin coding sequences [10]. The existence of such a large repertoire of MGEs in staphylococci is evidence of an open pan-genome with a constantly increasing collection of distinct genes [11–13]. This is exemplified in *S. aureus* and *S. epidermidis*, despite their core genome being limited and remarkably conserved, favoring their clonality. In contrast to sexual species such as *Streptococcus pneumoniae*, recombinations are very rare in *S. aureus*, and to a lesser extent in *S. epidermidis*. Similarly, Chassain et al. found that *S. lugdunensis* also presents a clonal population structure in multilocus sequence typing (MLST) studies with an allelic polymorphism even lower than in *S. aureus* and *S. epidermidis* [14, 15]. This observation, along with the highly conserved antibiotic susceptibility, probably indicates the existence of barriers to horizontal genetic transfer, and correlates with the difficulties experienced in transformation of *S. lugdunensis* [16–18].

Various genetic elements have been proposed that control genome stability in bacteria [19]. In *S. aureus*, whose genetic resistance to horizontal gene transfer (HGT) has long been noticed in laboratories, this relative “immunity” mainly relies on a strong restriction-modification (RM) system that also exists in CoNS such as *S. epidermidis*, and this noticeably impairs phage infectivity [20–23]. To date, four RM systems have been described in staphylococci—Types I, II, III, and IV—with Type II not observed in *S. epidermidis* [20, 24]. These systems comprise two enzyme factors, a restriction endonuclease and a methyltransferase, which may differ in their subunit composition, sequence recognition, cleavage position, cofactor requirements, and substrate specificity [24]. Heilbronner et al. showed that the *S. lugdunensis* strain N920143 possessed a functional Type I RM system (SluI), whose inactivation resulted in improved transformation with *E. coli* plasmid [16].

Clustered regularly interspaced short palindromic repeats (CRISPR) associated with Cas protein (CRISPR/Cas) systems have been described more recently in *S. aureus* and *S. epidermidis*, and constitute another strong barrier to foreign DNA uptake, particularly plasmid DNA [25–27]. In *S. aureus* and CoNS, Class 1 Type IIIA CRISPR/Cas systems have been predominantly identified, containing the universal *cas1–2* genes in addition to *cas6*, and *csm1* to *csm6* [28, 29]. Class 2 Type IIC CRISPR/Cas systems have also been identified in staphylococci, containing the *cas9* gene in addition to the *cas1–2* genes. Rossi et al. screened 122 genomes from 15 species of CoNS and found that only 15% of them harbored complete CRISPR/Cas systems, mainly from Type IIIA (Cas6-associated system) and Type IIC (Cas9-associated system) [28]. It has been proposed that this low abundance of CRISPR/Cas systems in CoNS (compared to other bacteria among which 40 to 50% bear CRISPR/Cas systems) could be linked to their role as gene reservoirs for other staphylococci such as *S. aureus*, particularly for antibiotic resistance genes [29].

Toxin/antitoxin (T/AT) systems form a third group of systems that might prevent foreign DNA uptake in bacteria, including *S. aureus* and CoNS. If, as has been proposed, their role in controlling bacterial growth and metabolic processes is central, then these systems could protect their host from phages and other MGE acquisition [30, 31]. Currently, various models have been described in *S. aureus*, including some among Type I systems (*SprA1/SprA1*_*AS*_, *SprF/SprG*), Type II systems (*MazEF*, *PemIK*, *YefM-YoeB*, *Omega/Epsilon/Zeta*), and Type III systems (*tenpIN*). *MazEF* was originally described in *E. coli* and was the first chromosomal T/AT system reported in *S. aureus*. Since then, a *MazEF* system has also been characterized in *S. equorum*, and several orthologues have been described in Gram-positive bacteria, but not in CoNS (other than *S. equorum*), even though the presence of such systems might be expected considering their wide distribution [32]. To date, the *MazEF* system from *S. aureus* is the best characterized, particularly through the work of Schuster et al. [33–36].

The extremely conserved antibiotic sensitivity profile of *S. lugdunensis*, along with the existence of various MGEs in this pathogenic species, motivated our study to explore its core and pan-genome profiles through comparative genomics analysis, and to further research the presence of barriers to HGT.

## Results

### Comparative genomics

**Fig. 1:**
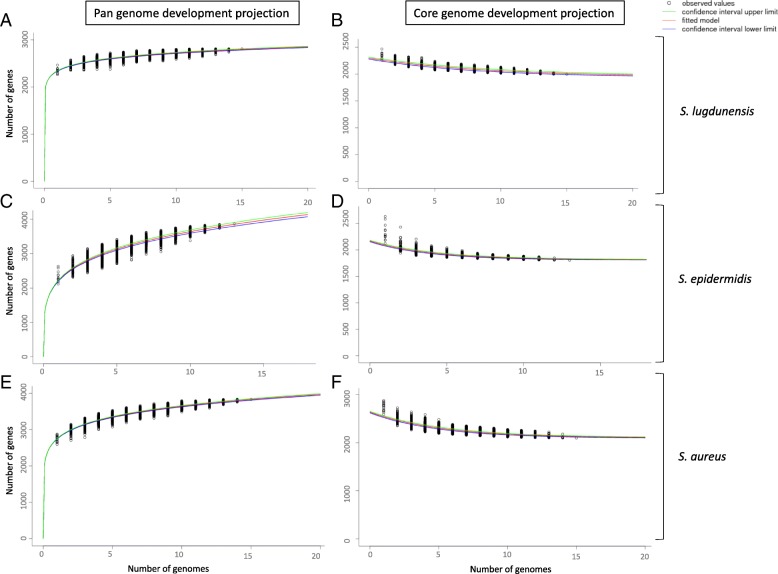
Pan-genome and core genome development plot projections for *S. lugdunensis* (Panel **a** and **b**)*, S. epidermidis* (Panel **c** and **d**)*, and S. aureus* (Panel **e** and **f**)*.* All calculations were done using the EDGAR software platform. For pan-genome development plot extrapolation: the red curve shows the fitted exponential Heaps’ low function, and the blue and green curves indicate the upper and lower boundary of the 95% confidence interval. For Core genome development plot extrapolation: the red curve shows the fitted exponential decay function, and the blue and green curves indicate the upper and lower boundary of the 95% confidence interval

The core and pan-genome development plots of *S. lugdunensis*, *S. aureus,* and *S. epidermidis* are shown in Fig. 1 and Additional file 1. *S. aureus* and *S. epidermidis* possess an open pan-genome that constantly grows as new genomes are added, reaching 3864 genes for *S. epidermidis* after the inclusion of 13 genomes, and 3828 genes for *S. aureus* after the inclusion of 15 genomes. In contrast, *S. lugdunensis* seems to possess a closed pan-genome that rapidly plateaus at under 3000 genes even after the inclusion of 15 genomes. The core genome of the 3 species displays a similar evolution, and rapidly stagnates at close to 2000 genes. Core and pan-genome development extrapolations gave the same results, projecting a constantly increasing number of genes in the pan-genome of *S. aureus* and *S. epidermidis* while projecting *S. lugdunensis* to plateau at under 3000 genes (Fig. 1 and Additional file 1). Growth exponent value was 0.066 (95% confidence interval 0.065–0.067) for *S. lugdunensis* versus 0.217 (95% confidence interval 0.214–0.220) and 0.123 (95% confidence interval 0.121–0.124) for *S. epidermidis* and *S. aureus,* respectively (Additional file 1). Core genome trends are similar, rapidly becoming limited to about 2000 genes for the three species. The extrapolated core genome sizes were 1944 (95% confidence interval 1933–1956) for *S. lugdunensis*, 2099 (95% confidence interval 2091–2015) for *S. aureus*, and 1811 (95% confidence interval 1807–1817) genes for *S. epidermidis*, respectively (Additional file 1). As suggested by MLST studies, *S. lugdunensis* might even possess a conserved core genome with average nucleotide identity (ANI) ranging from 99.5 to 99.9%, whereas it ranges from 97.5 to 99.8% for *S. aureus* and 96.6 to 99.7% for *S. epidermidis* (detailed results in Additional file 2) [14, 15]. Average amino acid identity (AAI) ranges were similar, from 99.5 to 99.9% for *S. lugdunensis*, 98.5 to 99.9% for *S. aureus* and 98.3 to 99.7% for *S. epidermidis*.

**Fig. 2:**
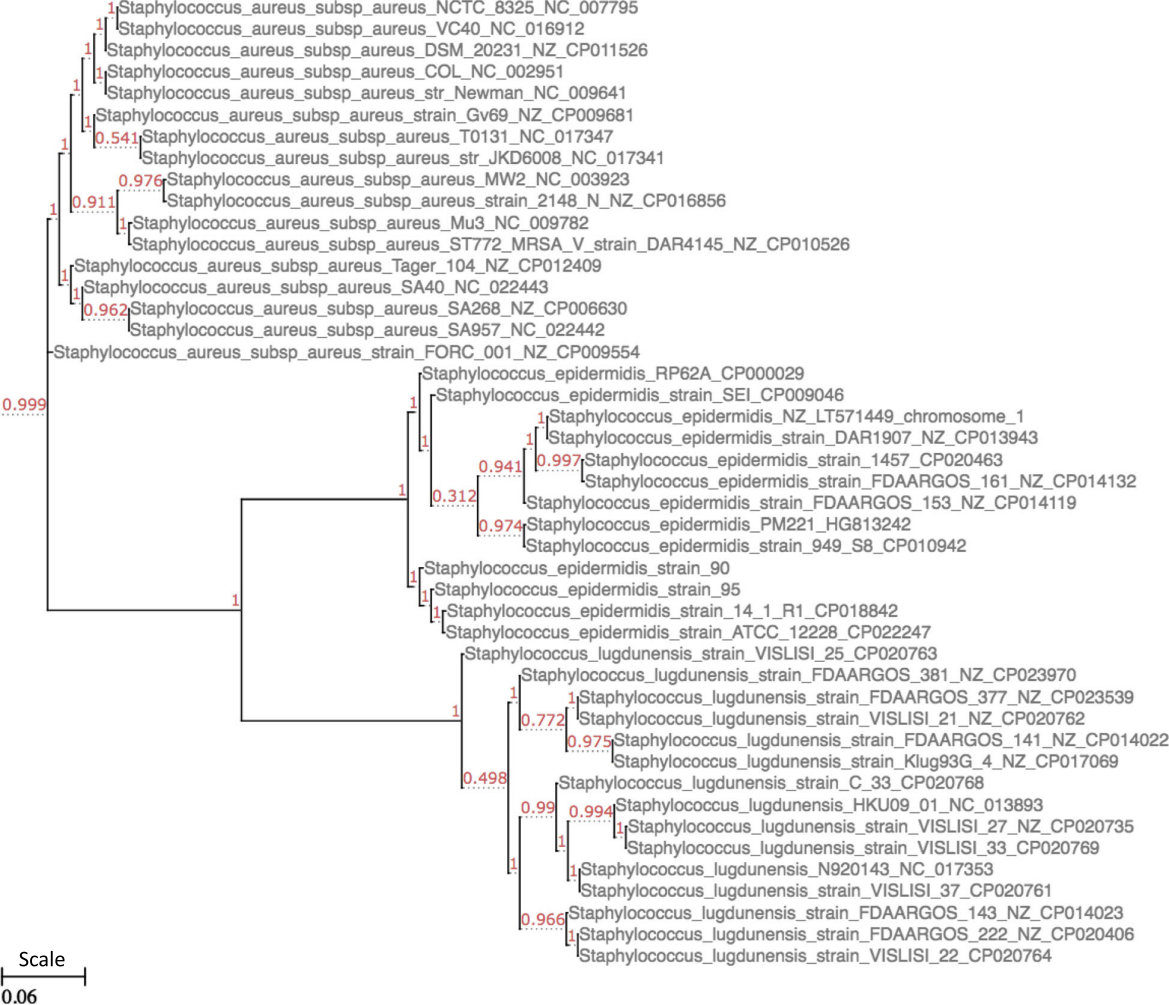
Phylogenetic tree illustrating genetic relationships between strains. The phylogenetic tree was built based on the complete core genome of the analyzed strains. Alignments of the each individual core gene set were generated using MUSCLE and subsequently concatenated to one large supermatrix. Fasttree was used to infer a maximum likelihood tree from this core gene alignment. Shimodaira-Hasegawa support values. Calculations were made for the three species and show that they form three clearly separated clusters within the phylogenetic tree

**Fig. 3:**
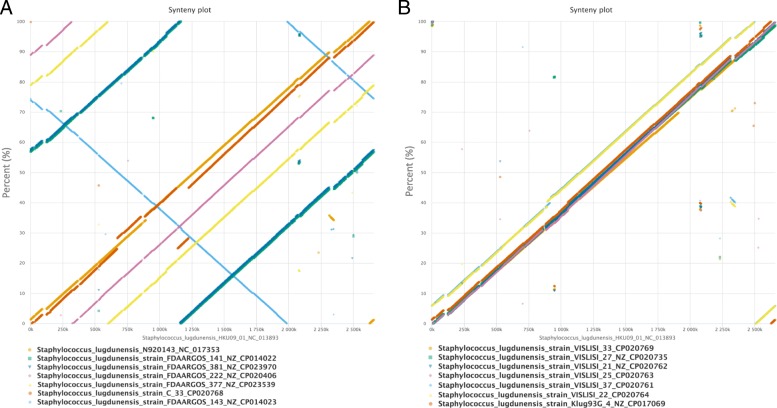
Structural genomic comparison of *S. lugdunensis* reference strain HKU0901 with the 14 other complete *S. lugdunensis* genomes. Synteny plots were produced by using EDGAR web server, showing the stop positions of orthologous gene pairs in different genomes. *S. lugdunensis* HKU09101 genome was compared with the 14 other genomes by splitting the analysis in two for better visual representation (panel **a** and **b**). Although some genomes have differing start positions, they all show a high degree of co-linearity, indicating a high level of relatedness and a low level of genomic rearrangement activity within the nalayzed set

A phylogenetic tree created for all three species together shows a clear separation of *S. aureus, S. epidermis,* and *S. lugdunensis* (see Fig. 2)*.* They all form monophyletic branches that are clearly separated from each other. Phylogenetic distances were very small in general, with the *S. lugdunensis* branch showing the lowest distances between within-species branches. Structural genomic analysis of *S. lugdunensis* genomes gave further evidence of the highly conserved genomes of the *S. lugdunensis* species. With the exception of small translocations in strains C33, VISLISI 37 and VISLISI 22, all 15 compared genomes show a highly conserved gene order, with no signs of larger genomic rearrangements. This again demonstrates the genomic stability of *S. lugdunensis* (Fig. 3).

### Functional analysis

**Fig. 4:**
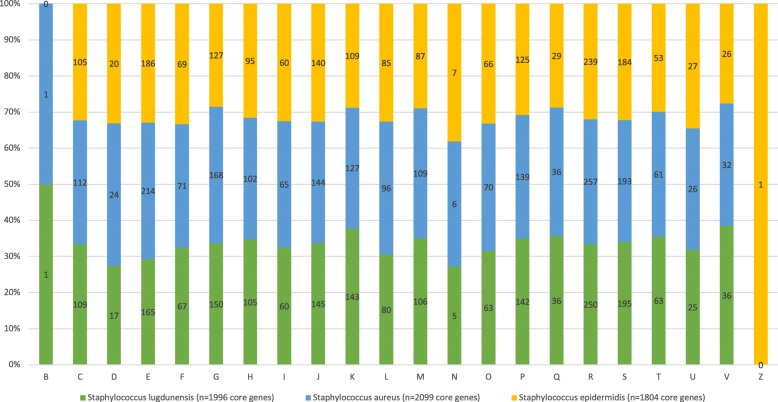
COG functional categories from the core genome of *S. lugdunensis*, *S. aureus,* and *S. epidermidis* strains. Gene lists were predicted using the EDGAR web server, and COG categories obtained by loading them into the WebMGA web server. COG categories are as follows: for cellular processes and signaling, **d** is cell cycle control, cell division, and chromosome partitioning; **m** is cell wall/membrane/envelope biogenesis; **n** is cell motility; **o** is post-translational modification, protein turnover, and chaperones; **t** is signal transduction mechanisms; **u** is intracellular trafficking, secretion, and vesicular transport; **v** is defense mechanisms; and **z** is cytoskeleton. For information storage and processing, **b** is chromatin structure and dynamics; **j** is translation, ribosomal structure, and biogenesis; **k** is transcription; and **l** is replication, recombination, and repair. For metabolism, **c** is energy production and conversion; **e** is amino acid transport and metabolism; **f** is nucleotide transport and metabolism; **g** is carbohydrate transport and metabolism; **h** is coenzyme transport and metabolism; **i** is lipid transport and metabolism; **p** is inorganic ion transport and metabolism; and **q** is secondary metabolite biosynthesis, transport, and catabolism. **r** is for general function prediction only, and **s** for unknown function

To compare core genome functional categories, we used functional assignments from the COG database. Results are shown in Fig. 4. The core gene category repartition was highly similar among the 3 species, exceptions being that *S. epidermidis* lacks any genes involved in chromatin structure and dynamics, and both *S. lugdunensis* and *S. aureus* lack any cytoskeleton category genes.

### Identification of barriers to HGT in MGEs

*S. lugdunensis* genome length ranged from 2.5 to 2.7 Mb, with GC content constituting between 33.7 and 33.9% (Table 1). All genomes contained 2397–2584 coding sequences, with 46–60 tRNA, 4–19 rRNA, and all strains displayed one tmRNA. In addition to the phages previously identified in the VISLISI strains, we identified 3 additional prophages. One additional plasmid was retrieved from the strain FDAARGOS_381. We did not identify pathogenicity islands in any of the 15 published genomes for *S. lugdunensis*. Seven complete prophages were identified (Table 2). Length ranged from 37.7–57 kb, and GC content varied from 33.8 to 35.2%. All are close to known prophages previously identified in *S. aureus*, *S. epidermidis,* and *S. hominis*. Four of the 7 prophages exhibited a Zn^2+^ carboxy peptidase gene sequence, but no sequences for antibiotic resistance genes or T/AT systems. We identified a CRISPR-associated gene *cas2* in the phage from VISLISI_22, but without either CRISPR-associated genes or CRISPR sequence. None of the plasmid sequences retrieved from the GenBank database carried any loci coding for protease, PIN-like domain, T/AT, or CRISPR/Cas. A Type II RM system was identified in C33 pVISLISI_5, and sequence analysis is detailed below.

**Table 1:** *S. lugdunensis* whole genome sequence content in comparison with *S. aureus* and *S. epidermidis*

	Strain	Size (Mb)	GC (%) Content	Gene	Protein	rRNA	tRNA	tmRNA	Plasmids	Phages	PRCIs^1^
*S. lugdunensis*	HKU0901	2.7	33.9	2567	2425	19	61	1	0	1	0
	N920143	2.6	33.8	2498	2383	16	55	1	0	1	0
	FDAARGOS_141	2.6	33.8	2465	2350	19	60	1	0	0	0
	FDAARGOS_143	2.6	33.9	2515	2347	19	60	1	0	1	0
	FDAARGOS_222	2.5	33.8	2414	2261	16	59	1	0	0	0
	Klug93G-4	2.6	33.8	2501	2358	20	69	1	0	0	0
	FDAARGOS_377	2.6	33.8	2479	2351	19	60	1	0	0	0
	FDAARGOS_381	2.6	33.8	2482	2344	19	60	1	1	0	0
	VISLISI_21	2.5	33.7	2437	2344	6	46	1	0	0	0
	VISLISI_22	2.6	33.8	2459	2353	6	59	1	1	1	0
	VISLISI_25	2.5	33.8	2397	2293	4	48	1	0	0	0
	VISLISI_27	2.6	33.7	2508	2391	7	59	1	1	0	0
	VISLISI_33	2.7	33.7	2584	2465	6	55	1	1	2	0
	VISLISI_37	2.6	33.7	2482	2391	6	52	1	0	1	0
	C33	2.5	33.9	2405	2292	5	52	1	2	0	0
*S. aureus*	MW2	2.8	32.8	2934	2778	19	60	1	0	2	1
*S. epidermidis*	ATCC12228	2.6	32.0	2545	2378	19	60	1	2	0	1

1 Phage Related Chromosomal Islands (including *S. aureus* pathogenicity islands)

**Table 2:** *S. lugdunensis* complete prophages and identification of putative barriers to HGT

Strain	Complete Prophage	Length (kb)	GC%	Total Proteins	Common Phage	Peptidases	T/AT^1^	RM^2^	CRISPR/Cas Systems
HKU0901	1	37.7	34.57	58	PHAGE_Staphy_PH15^3^	0	0	0	0
N920143	1	49.4	34.42	63	PHAGE_Staphy_TEM123^4^	Zn2+ carboxy peptidase	0	0	0
FDAARGOS_143	1	57.0	35.24	55	PHAGE_Staphy_CNPx_NC_031241^5^	0	0	0	
VISLISI_22	1	49.4	34.3	53	PHAGE_Staphy_StB12^6^	Zn2+ carboxy peptidase	0	0	*cas2*
VISLISI_33	1	44.4	33.8	52	PHAGE_Staphy_StB12	Zn2+ carboxy peptidase	0	0	0
	1	28.1	34.4	52	PHAGE_Staphy_PH15	0	0	0	0
VISLISI_37	1	47.0	34.35	58	PHAGE_Staphy_StB12	Zn2+ carboxy peptidase	0	0	0

^1^ T/AT systems

^2^ RM systems

^3^ PHAGE_Staphy_PH15: *S. epidermidis* phage

^4^ PHAGE_Staphy_TEM123: *S. aureus* phage

^5^ PHAGE_Staphy_CNPx_NC_031241: *S. epidermidis* phage

^6^ PHAGE_Staphy_StB12: *S. hominis* phage

### Identification of CRISPR/Cas systems

**Fig. 5:**
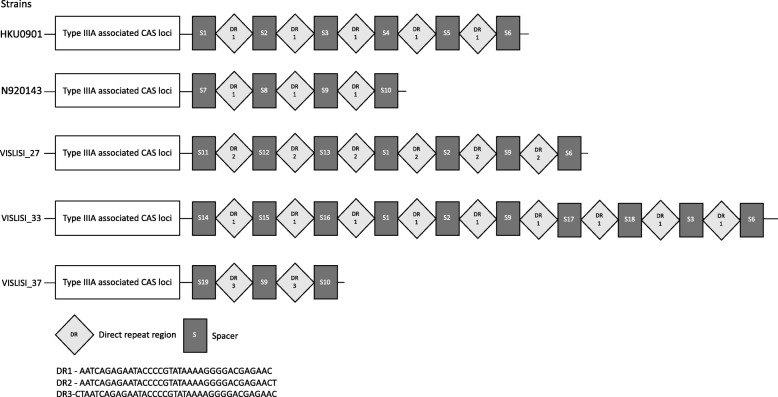
DR sequences and spacers in type IIIA CRISPR/Cas systems from *S. lugdunensis*

CRISPRFinder identified several CRISPR structures in the 15 *S. lugdunensis* genomes, some being confirmed CRISPR sequences, others being questionable as CRISPR either because of their small size (with only 2 or 3 direct repeat (DR) sequences), or because the repeat motifs in the CRISPR were not 100% identical. The complete list of all CRISPRs recovered is available in Additional file 3. Overall, 6 confirmed CRISPR/Cas systems were identified in 6 different genomes from the strains: HKU0901, N920143, VISLISI_27, VISLISI_33, VISLISI_37, and C33. Several questionable CRISPRs were identified in all 15 strains, with a total number ranging from 4 to 11 per genome. The genetic environment of all CRISPR sequences was analyzed using ARTEMIS (v.16.0.0), and we identified Cas genes in association with the 6 confirmed CRISPR sequences. The CRISPR/Cas systems from HKU0901, N920143, VISLISI_27, VISLISI_33, and VISLISI_37 corresponded to a Class 1 Type IIIA system according to the classification of Koonin et al., with the conserved modular organization of this family [29]. The adaptation module comprised *cas1* and *cas2*, followed by the small subunit loci *csm1* to *csm6*, the *cas6* gene, which is the endonuclease that belongs to the effector module, and finally the CRISPR sequences. Unlike in *S. aureus*, the CRISPR sequences are located downstream of the *cas6* gene, as seen in most other Type IIIA CRISPR/Cas systems in CoNS [26, 28]. These 5 CRISPR/Cas systems were aligned using Easyfig (v.2.2.2), and showed 100% sequence identity regarding the Cas coding sequences (Additional file 4). The CRISPR regions showed variable sequence identity levels, ranging from 71 to 100%. We identified 19 distinct spacers and 3 DRs (Fig. 5). Spacer sequence details are available in Additional file 3. No known origin was found in BLAST for any of the 12 spacers, whereas putative matches were found for 7 of them with sequences that might originate from known MGEs. Results are detailed in Table 3. We also observed that DRs are highly conserved and nearly identical in all 5 strains. In particular, the core region included a CCCC region separated by 8 nucleotides from a GGGG pattern; these could interact to form the typical hairpin structure involved in the initial processing of the CRISPR transcript. This conserved motif is also present in other CoNS and *S. aureus* [26, 28].

**Table 3:** Origin of the spacers of the 5 Type IIIA CRISPR/Cas systems from *S. lugdunensis* strains HKU0901, N920143, VISLISI_27, VISLISI_33, and VISLISI_37

Spacers	BLAST Match with Known MGEs	Cov^1^	ID^2^	Score^3^
S1	*Campylobacter* phage CP220	81%	90%	41
S2	*Clostridium botulinum* plasmid pND7	74%	92%	39
S4	*Lactobacillus* plasmid	78%	92%	37
S5	*Bacillus thuringiensis plasmid* pAM65–52-2-350 K	87%	85%	39
S6	*Lactobacillus salivarius* ZL5006 plasmid	77%	89%	37
S7	*Streptococcus* phage IPP55	77%	93%	39
S15	*Staphylococcus* phage vB_SepS_SEP9	100%	89%	48
S3, S8–14, S16–19	None			

^1^ Coverage level

^2^ Identity level

^3^ BLAST score

Unlike in the other 5 strains, the C33 complete CRISPR sequence was not associated with Type IIIA Cas coding loci but with Class 2 Type IIC *cas* genes (as classified according to Koonin et al. [29]), including the *cas1* and *cas2* genes from the adaptation module, and the *cas9 gene,* which is the effector of this CRISPR/Cas system type. CRISPR sequences were located upstream of the *cas9* gene that displayed several stop codons, making it a pseudogene and the whole operon probably ineffective. Nevertheless, when analyzing the possible BLAST matches of the 11 spacers, only 1 match for the second spacer was observed, for a *Bacillus* phage sequence (coverage 66%, identity 100%, score 39).

Finally, we performed BLAST searches for the 91 questionable CRISPR sequences that were not associated with any *cas* loci, since several CRISPRs described in the literature are not associated with *cas* genes, and conversely, several *cas* genes might be isolated [37]. We identified one recurrent DR sequence that does not match any of the 3 DRs identified in the Type IIIA complete CRISPR/Cas systems of *S. lugdunensis*, and that notably lacks the highly conserved CCCC-GGGG motif, which might confer loss of function. Nevertheless, we also identified 1 recurrent spacer that gives an interesting BLAST hit with the *S. aureus* pathogenicity island SaPI2 from the strain RN3994 (coverage 100%, identity 100%, score 79) [38]. Thus, most of these sequences are probably real orphan CRISPRs that have lost their function and are not misidentified repeated sequences (false CRISPRs).

### Identification of T/AT systems

T/AT identification was performed on the available annotations of the 15 annotated *S. lugdunensis* genomes, and on the de novo annotations that were generated using PROKKA (v1.12). As described for *S. equorum* and *S. aureus*, we identified a complete *MazEF-rsbUVW-sigB* T/AT system in the 15 strains with a conserved operon organization at about 100% BLAST identity (Additional file 5). The *mazEF* genes are located upstream the of the *sigB* locus that comprises *rsbU*, *rsbV*, *rsbW* and *sigB*. We also identified an alanine racemase *rac* that belongs to this operon (whose role has not been clearly determined to date). MazEF has been reported in the genomes of several Gram-positive bacteria, but among these, the only CoNS representative is *S. equorum* KM1031 (NCBI accession number NZ_CP013980.1). We therefore extended the search for this system in *S. aureus* MW2, and 3 other CoNS: *S. xylosus* strain S170 (NCB accession number NZ_CP013922.1), *S. capitis* FDAARGOS_378 (NCBI accession number NZ_CP023966.1), and *S. epidermidis* strain ATCC12228 (NCBI accession number) (Additional file 5). All genes from the MazEF operon of the 15 *S. lugdunensis* genomes have conserved open reading frames except the strain FDARRGOS_143 in which the MazF coding sequence consisted of nonsense mutations.

**Fig. 6:**
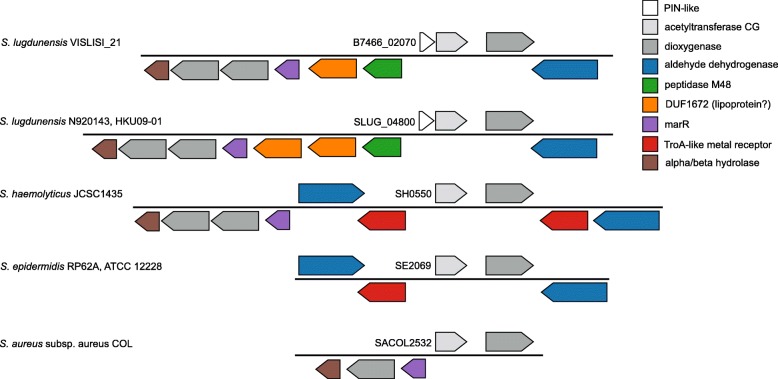
Genomic context of the PIN-like domain coding sequence of *S. lugdunensis* and other staphylococci

We did not identify any of the other T/AT systems that have been previously described in *S. aureus* as detailed in the material and methods section. Nevertheless, we found one locus with a predicted PIN-like domain in 13 of the 15 *S. lugdunensis* genomes. This locus was systematically associated with 1 metalloprotease coding sequence that might belong to the M48 family (according to the MEROPS database), and 1 N-acetyl transferase coding sequence, leading us to hypothesize the presence of a possible T/AT system (Fig. 6) [39]. These 3 loci, and the 10 kb upstream and downstream nucleotide sequences, display 100% sequence identity in the 13 genomes. The PIN-like domain locus could be regarded as a pseudogene rather than a functional PIN gene, since the length of its coding sequence was 41 amino acids, while the minimum length for the PIN fold is ~ 100 amino acids. Also, its protein product lacks a cluster of positively charged residues that would be necessary for nucleic acid binding, and consequently might fail to work as a functional nuclease, as expected for PIN-domain containing proteins. The downstream gene encodes a member of the minimal acetyltransferase CG family (GCN5-related N-acetyl-transferase, Pfam family PF14542). In an article reporting its crystal structure in an *S. aureus* member of this family, it was suggested that a second protein providing a substrate-binding region must combine with it to yield fully functional N-acetyltransferase [40]. It could be that in *S. lugdunensis* the binding partner for the GNAT-like protein is the deteriorated PIN protein, and the acetylation target could be the MarR-like (of the HTH fold) protein, encoded on the other strand.

### Identification of RM systems in the main chromosome of *S. lugdunensis.*

All 15 genomes were examined for the presence of RM systems using the REBASE database, resulting in the identification of multiple Type I and Type II RM systems (Table 4 and Additional file 6 for genomic coordinates) [23],. Six nearly identical Type I RM systems were identified in HKU0901, N920143, FDAARGOS_143, VISLISI_27, VISLISI_33, and VISLISI_37. The methylase coding sequence displayed 90 to 98% aminoacid identity level with the methylase *Sau*18 from *S. aureus* strain C18, a draft *S. aureus* genome with 123 contigs (NCBI accession number GCA_001921685.1). The methylase target specificity remains unknown. All 6 operons comprised 3 consecutive genes as described for this RM Type: *hsdR* (restriction locus), *hsdM* (modification locus), and *hsdS* (specificity locus). A BLAST alignment performed with Easyfig (v.2.2.2) found nearly 100% sequence identity among all 6. We extended the comparison with *S. aureus* strain MW2, and with *E. coli* strain K-12 (GenBank accession number SC000913.3), which bears the canonical Type I RM, *Eco*KI [41]. We found a very low level of identity between *Eco*KI and *S. lugdunensis* VISLISI_33 *hsd* subunit amino acid sequences, with identity scores ranging from 16 to 25% (according to EMBOSS Needle pairwise sequence alignment). Conversely, we observed higher levels of identity between *S. lugdunensis* VILSISI_33 and *S. aureus* MW2 loci, with scores of 71, 58, and 35% for *hsdM*, *hsdR,* and *hsdS*, respectively. Alignment files are available in Additional file 7. Interestingly, despite low AAI levels between *hsdR* from *S. aureus* and that from *S. lugdunensis*, we observed that the essential motifs for DNA cleavage and translocation were highly conserved according to the amino acid sites identified by Roberts et al. This could support the hypothesis that both systems belong to the same subfamily [41]. The strains FDAARGOS_143 and VISLISI_27 have 2 other distinct Type I RM systems, with their methylase coding sequences displaying 97% AAI level with methylase *Sca*9557 from *S. caprae* strain C18, and 94% AAI level with *Sau*MSSIII from *S. aureus*, respectively. Type II RM systems were identified in 4 strains, FDAARGOS_222, VISLISI_22, VISLISI_25, and the plasmid sequence pVISLIS_5 from C33. pVISLISI_5 is a mobilizable plasmid with a *repA* replication gene, whose closest homologous plasmid is VRSAp from *S. aureus* (NCBI accession number NC_002774.1) [5]. The closest homologue found for the methylase of pVISLISI_5 (60% identity) was *Efa*PC41 from *Enterococcus faecium* strain PC4.1 (and there are no RM systems in VRSAp). The methylase from FDAARGOS_222 and VISLISI_22 showed 82% identity with *Sha*J from *S. haemolyticus*, and the nucleotide sequence specificity was known (GATC). Finally, the Type II RM system from VISLISI_25 was unique among *S. lugdunensis* strains. The methylase gene was duplicated as seen in its closest homologue, *Sep*60 from *S. epidermidis*, and the sequence specificity was known (GGTGA).

**Table 4:** RM systems identified in *S. lugdunensis* using the REBASE database, and homology analysis of methylase

*S. lugdunensis*	RM System	Closest Methylase				
		ID^1^	ID score	REBASE score	Strain origin	Nucleotide specificity
HKU0901	Type I	M.*Sau*C18	98%	1127	*S. aureus* C18	Unknown
N920143	Type I	M.*Sau*C18	91%	1128	*S. aureus* C18	Unknown
FDAARGOS_141	None					
FDAARGOS_143	Type I	M.*Sau*C18	90%	1121	*S. aureus* C18	Unknown
	Type I	*Sca*9557	97%	1170	*S. caprae* 9557	Unknown
FDAARGOS_222	Type II	M.*Sha*J	82%	844	*S. haemolyticus* JCSJ1435	GATC
VISLISI_22	Type II	M.*Sha*J	82%	844	*S. haemolyticus* JCSJ1435	GATC
VISLISI_25	Type II	Methylase 1				
		M2.*Sep*60	66%	329	*S. epidermidis* BCM-HMP0060	GGTGA
		Methylase 2				
		M1.*Sep*60	66%	558	*S. epidermidis* BCM-HMP0060	GGTGA
VISLISI_27	Type I	M.*Sau*C18	91%	1128	*S. aureus* C18	Unknown
	Type I	M.*Sau*MSSIII	94%	1346	*S. aureus* MSSA476	TAAYNNNNNNNTCNNC
VISLISI_33	Type I	M.*Sau*C18	91%	1128	*S. aureus* C18	Unknown
VISLISI_37	Type I	M.*Sau*C18	91%	1128	*S. aureus* C18	Unknown
C33 pVISLISI_5	Type II	M.*Efa*PC41	60%	312	*E. faecium* PC4.1	Unknown

^1^ Identity

## Discussion

This study presents the first comparative genomic analysis of *S. lugdunensis*, a species emerging as a significant nosocomial pathogen [3]. Pan-genome and core genome analyses revealed that *S. lugdunensis* displays a closed pan-genome in contrast to all other staphylococci studied to date and to most commensal and pathogenic human bacteria [11, 42–46]. This wholly unexpected observation could be explained by the concomitant identification of several barriers to HGT, namely, CRISPR/Cas, RM, and T/AT loci that constitute specialized systems preventing HGT, particularly through MGEs. Although RM systems are widespread in staphylococci, according to the REBASE database, the identification of T/AT systems in 100% of the 15 *S. lugdunensis* genomes, and of complete CRISPR/Cas systems in 33% of them, is more surprising. Indeed, in 2017 Rossi et al. found that only 15% of 122 genomes in 15 different CoNS species harbored complete CRISPR/Cas systems [28]. In addition, T/AT systems have been described in only 1 CoNS species, *S. equorum.* The characterization of multiple HGT prevention systems in a single species, *S. lugdunensis*, is consistent with the presence of a closed pan-genome. Besides specialized elements such as phages and plasmids, homologous recombination constitutes another frequent modality for HGT, but it is seldom seen in staphylococci, even though such a mechanism may have had an impact on the evolutionary history of lineage separation. Indeed, Meric et al. found evidence that homologous recombination might have changed 40 and 24% of the core genome of *S. epidermidis* and *S. aureus*, respectively [13]. However, over a short time scale, such events are extremely rare, and the core genome remains mostly conserved. The high values we found for AAI and ANI from *S. lugdunensis* genomes, higher even than for *S. epidermidis* and *S. aureus*, also suggest that genomic diversity of this species is lower than for other staphylococci, and this observation clearly correlates with the previously reported highly clonal population structure of this species [14, 15].

MGEs are able directly and rapidly (within hours, even) to modify the *Staphylococcus* accessory genome in vivo via any genetic exchange occurring between *S. aureus* and *S. epidermidis* [7]. MGEs are not to be underestimated in their ability to reshape the whole bacterial genome, even in what are usually considered as “immune” species, such as staphylococci (which display a small genome size that reflects evolutionary constraints that probably fitted them to a limited number of hosts). Interestingly, a pan-genome study of the sexual species *S. pneumoniae*, which is highly susceptible to HGT through homologous recombination, showed a relatively limited pan-genome size that plateaued at under 5000 genes, placing this species on the boundary between an open and closed pan-genome [43]. The openness of the pan-genomes of *S. aureus* and *S. epidermidis* obviously relies on MGEs and, if their core genome is highly conserved, their dispensable genomes offer an extremely large repertoire of genes that confer specific advantages in a defined host under particular environmental conditions, and from a clinical point of view, support their virulence [11]. Additionally, in staphylococci, MGEs allow the occurrence of inter-species genetic exchange, providing real potential for CoNS to act as gene reservoirs facilitating the transfer of methicillin resistance to *S. aureus*, especially since *S. aureus* has recently been exposed as a putative gene reservoir for CoNS [47, 48]. In this context, the existence of a closed pan-genome in *S. lugdunensis* (a species emerging as a significant pathogen) and a putative relative immunity to HGT cannot be clearly understood in terms of evolutionary advantage. MGEs facilitate the acquisition of genes conferring antibiotic resistance and thus confer evolutionary advantage in staphylococci such as *S. epidermidis* and *S. aureus,* which easily and frequently acquire various resistance genes (one example being methicillin resistance through SCC *mec*). However, *S. lugdunensis* remains highly susceptible to most antibiotics, and identification of SCC *mec*-bearing strains is rare; another illustration of its apparent immunity to HGT.

Another hypothesis could be that *S. lugdunensis* speciation has occurred only recently, and we are only now experiencing the start of the emergence of new *S. lugdunensis* clones whose genomes have been augmented by various MGEs originating from other CoNS or *S. aureus*; and yet, *S. lugdunensis* has been studied for several years now, even in clinical settings where such genetic exchange should have occurred. In addition, our study included genomes from strains originating not just from one location but from multiple countries and various settings (nosocomial, community, infective, and contaminant strains) (see Additional file 8). Since its first description in 1988 by Freney et al., several phylogenetic studies have suggested that *S. lugdunensis* always appears to occupy a unique cluster group, whatever the method used for phenotyping (16S rRNA, housekeeping genes, whole genome sequences) [49–51].

The role of the MazEF T/AT system in *S. lugdunensis* has to be interpreted in the light of its particular location on the bacterial chromosome. If the roles of plasmid T/AT systems have been only partially elucidated, those of chromosomal T/AT are even less well understood (Fernández-García et al. 2016; Lee & Lee 2016; Schuster & Bertram 2016) [33, 49, 50]. It has been suggested that such elements lead to genetic stabilization of various MGEs as prophages or pathogenicity islands, or impact the stress response functions of modular elements of bacterial growth and death [49, 51]. Interestingly, Saavedra De Bast et al. also showed that chromosomal T/AT systems could efficiently act as anti-addiction modules by protecting bacteria against post-segregation killing, a mechanism by which plasmid-encoded T/AT systems favour plasmid maintenance by eliminating daughter bacteria that do not receive a plasmid copy [30]. The widespread occurrence of the MazEF system and its highly conserved nucleic acid sequence do not support the hypothesis that it is a simple remnant of past evolutionary events, and its role needs now to be phenotypically elucidated. By searching T/AT systems, we identified a PIN-associated locus with an undetermined role, although the genetic environment might help us to formulate a hypothesis. Perhaps the PIN protein used to work as a toxin in a toxin-antitoxin system, as a partner for another helix-turn-helix (HTH) folded transcription factor. HTH transcription factors are typical antitoxins for PIN-like toxins. The MarR protein could work as a transcription factor, regulating transcription of other genes involved in that pathway. However, the system could also be independent of the PIN protein, since the homologous operon is absent in other *Staphylococcus* species (Fig. 6), and acetylation could be performed on a metabolite or an antibiotic [52]. Other genes in the genomic neighborhood would support this hypothesis; they encode dioxygenases, aldehyde dehydrogenases, alpha/beta hydrolases and some metal-binding receptors, which could work in a concerted way to detoxify a specific molecule.

Regarding CRISPR/Cas loci, we identified a complete Type IIIA CRISPR/Cas system in 5 strains among the 15 studied, whereas such systems have been identified in only 15% of CoNS. CRISPR/Cas systems can efficiently prevent plasmid conjugation and transformation, as well as phage infection. This specific observation has been reported in *S. epidermidis* and *S. aureus* Type IIIA CRISPR/Cas systems [26]. Our genetic findings need functional confirmation, but a similar observation with *S. lugdunensis* would be expected. Additionally, we identified several orphan CRISPR sequences with a repeated spacer that might correspond to an extract from the sequence of *S. aureus* pathogenicity island SaPI2. The significance of such sequences is unknown, but they probably constitute remnants of past genomic events involving MGEs, and their role in *S. lugdunensis* might be limited or even non-existent due to the absence of functional DR sequences. Isolated CRISPRs can be orphans, though it has been shown that they may be functional in combination with distant *cas* loci, even where the median distance between CRISPR and corresponding type *cas* genes is 268 bp for type IIIA, and 103 bp for Type II [37]. Additionally, questionable CRISPRs can also be false CRISPR sequences, corresponding to other kinds of repeated element such as tandem repeats, *S. aureus* repeat (STAR) elements, or even simple repeat elements [53, 54]. Regarding isolated *cas* loci, they are widespread in bacterial genomes as remnants of lost CRISPR/Cas complete systems, now without any immune function; however, they could play a role in the maintenance of other functions, such as DNA repair [37].

Finally, we identified several Type I and Type II RM systems in 10 strains among the 15 studied, and 1 Type II RM system in a plasmid sequence. RM systems have many features in common with T/AT systems, one being cell killing in the case of foreign DNA invasion which, in this case, is based on epigenetic identities (methylation level) [55]. RM systems, particularly Type I, are one of the major mechanisms by which *S. aureus* prevents HGT. Phage- and plasmid-mediated HGT between *S. aureus* strains from different lineages is strictly controlled by RM systems, particularly Type I [41]. Identification of such systems in CoNS is exceptionally rare; we found only one complete report involving the presence of an RM system in *S. epidermidis* [20]. The presence of an RM system in *S. lugdunensis* constitutes a novel barrier for HGT, a situation identified by Heilbronner et al. as the main obstacle for transformation with *E. coli* using a *hsdR* mutant [16].

We reported in a previous study the whole genome sequence of seven *S. lugdunensis* strains and the presence of MGE: plasmids and prophages which genetic content suggested the existence of HGT with CoNS and *S. aureus* [5]. The results of the present study suggest that such HGT might remain scarce and, if they can mobilize genetic elements between those species, and enrich the whole genome, they are probably too rare to enrich significantly *S. lugdunensis* pan-genome.

Our study is limited by the number of *S. lugdunensis* genomes included even if pan and core genome size extrapolation tool that uses a Heaps’ law function, gave concordant results. Among 15 *S. lugdunensis* genomes, seven originated from a unique location over a 3 year period (VISLISI clinical trial), which might have limited, de facto, the genetic diversity of the genomes. In addition, we did not bring any evidence of a causative link between the presence of several barriers to HGT and a closed pan-genome, we only observed their co-occurrence which is only very suggestive.

## Conclusions

*S. lugdunensis* displays a closed pan-genome, a striking observation for a human pathogenic bacterium, and particularly for a *Staphylococcus*. This trait is co-occurring with the presence of multiple and dispersed mechanisms that could prevent HGT by MGEs then suggesting their implication in such an unusual pan-genome profile. Functional analysis using knockout mutants is now needed to prove that all the described operons are operational. Also, the presence of such systems in *S. aureus*, and also more rarely in other CoNS that display an open pan-genome, lead us to hypothesize the existence of other mechanisms. Identification of PIN-like domain-encoding loci, and of several putative nucleases, constitute new pathways that need to be explored.

## Methods

The 15 *S. lugdunensis* genome sequences used in this study, and their associated plasmid sequences, were taken from the GenBank database. *S. lugdunensis* strain HKU0901 (NCBI accession number NC_013893), whose complete genome sequence was first published in 2010 by Tse et al., was used as a reference in the comparative analyses [56]. Clinical and geographical origins of all 15 strains are listed in Additional file 8. Regarding *S. aureus*, we randomly selected 1 genomes from the 299 complete sequences in the GenBank database, where 8450 genome assemblies are available, most being draft sequences. The genome of *S. aureus* strain DSM 20231 (NCBI accession number NZ_CP011526), served as a reference in the comparative analyses. The complete genome of this type strain was determined in 2015 by PacBio single-molecule real-time technology by Shiroma et al., and proposed as a reference strain to perform comparative genomic studies due to its genotypic and phenotypic characteristics [57]. Thirteen complete genomes of *S. epidermidis* among the 532 genome assemblies available in the GenBank database were included in this study (a fourteenth genome from strain GTH 12 is also available but with no annotation). *S. epidermidis* strain ATCC_12228 (NCBI accession number NZ_CP022247), a non-biofilm-forming, non-infection-associated strain, was selected as a reference for the comparative analyses. NCBI accession numbers of all strains are listed in Additional file 8. We excluded draft sequences from *S. lugdunesis* (eight additional genomes) and *S. epidermidis* (518 additional genomes) for which only scaffolds are available and no finished bacterial chromosome. Those genomes might present several gaps, no or uncomplete annotations, and no error correction steps during assembly process. The use of draft genomes in comparative genomics is questionable, even not recommended, particularly for synteny studies, and we favored stringency in this study. Every gap in draft sequences may split, truncate or completely mask a gene which may add bias to the EDGAR software platform analyses [58, 59].

### Whole genome sequence analysis and comparative genomics.

Identification of the core genome and pan-genome was performed using the EDGAR software platform [59]. Several tools have been made available recently as stand-alone, open source, or web-based tools [60]. EDAGR 2.2 is a powerful tool that uses several publicly available genomes but also accommodates customized projects and genomes. The orthology analyses for pan-genome and core genome calculations in EDGAR are performed using BLAST score ratio values (SRV) with an orthology threshold calculated from the analyzed data rather than a fixed cut-off [61]. All calculations are made starting with 1 reference genome. The software allows calculation of pan genome and core genome subsets, as well as statistical extrapolation of core- and pan-genome sizes for a larger number of genomes.

For the statistical extrapolation, it uses non-linear least-squares curve fitting of the observed core and pan genome sizes as function of the number of analyzed genomes. For the core genome extrapolation an exponential decay function is used as described by Tettelin et al., where *c* is the amplitude of the function, *n* is the genome number, Ω is the extrapolated size of the core genome for *n* → ∞, and τ is the decay constant indicating the speed at which *f* converges to Ω [62].

$$ f(n)=c\cdotp \mathit{\exp}\left(-{n}^{\tau}\right)+\varOmega $$

For the pan genome, a Heaps’ power law function is used, where *n* is the number of compared genomes, *c* is a proportionality constant and γ the growth exponent that indicates at which speed the pan genome is growing [63].

$$ f(n)=c\cdotp {n}^{\gamma } $$

Results were compared among *S. lugdunensis*, *S. aureus,* and *S. epidermidis*.

For EDGAR phylogeny analyses, the pipeline uses the complete core genome. Every set of orthologous genes found in all genomes are separately aligned using the multiple alignment tool MUSCLE phylogenetic tree using the maximum likelihood approach as implemented in Fasttree 2 [64]. The trees calculated by Fasttree provide local support values calculated using the Shimodaira-Hasegawa test [65]. The phylogenetic trees were produced from newick file by using Phylogenetic Tree Viewer from ETE Toolkit [66]. Synteny and rearrangements in *S. lugdunensis* genomes were explored by using EDGAR. *S. ludgunensis* strain HKU0901 were chosen as a reference to create synteny plots. ANI and AAI matrices were calculated for the 3 species using the EDGAR interface. For AAI matrix calculation, all needed sequence similarity information is available from the BLAST step underlying the EDGAR orthology estimation method. Average nucleotide identity values are computed as described by and as implemented in the popular JSpecies package [67, 68].

### Functional analyses

Functional categories of the putative proteins encoded by *S. lugdunensis*, *S. aureus,* and *S. epidermidis* were compared using the clusters of orthologous groups of proteins (COG) database. COG categories were retrieved using the WebMGA software platform, with an e-value cut-off of 0.001 for prediction [69, 70]. We compared the putative functions of the proteins encoded by the core genome of these three species as issued by EDGAR.

### Identification of MGEs

MGEs from *S. lugdunensis* were searched to identify elements potentially controlling genome stability (such as CRISPR/Cas and T/AT systems). From previous studies, we had characterized several MGEs (prophages and plasmids) in *S. lugdunensis* strains coming from the VISLISI clinical trial [3, 5], and these were retrieved first. The analysis was extended to all complete *S. lugdunensis* genomes available in the GenBank database with the same methodology [5]. Briefly, prophage searches and annotations were performed using PHASTER (Phage Search Tool Enhanced Release) [71]. Plasmids were retrieved from GenBank database. Pathogenicity islands were identified through IslandViewer4 [72].

### Identification of CRISPR/Cas systems in *S. lugdunensis*

Several tools exist for CRISPR/Cas identification in whole genome sequences [73]. CRISPRFinder is a web server that offers a regularly updated database of CRISPR sequences that may be searched within an entire genome [74]. It does not focus on the genetic environment of the CRISPR sequences, and thus it does not identify the *cas* genes. To do this, we loaded the annotations from the GenBank database and the PROKKA de novo annotated genomes into Artemis software (v.16.0.0) to retrieve the CRISPR sequences identified with CRISPRFinder, and to analyze their genetic context [75]. All open reading frames surrounding the CRISPR sequences were also manually verified using the Uniprot and BLAST databases. We set the limits to 15 kb upstream and downstream of the CRISPR sequences, since *cas* genes were expected to be found in very close proximity, and because CRISPR/Cas system operons are not expected to be larger than 15 kb, particularly type IIIA and II [26, 28, 29]. All CRISPR/Cas sequences identified were aligned using Easyfig (v.2.2.2) to generate a BLAST alignment figure, with a minimum length of 100 bp, maximum e-value of 0.001, and minimum identity value of 90 [76]. CRISPR spacer origins were analyzed using the BLAST and Uniprot databases to search for known homologies.

### Identification of T/AT systems in *S. lugdunensis*

To identify T/AT systems in *S. lugdunensis,* we loaded the GenBank annotated genomes and the PROKKA de novo annotated genomes into the Artemis software (v.16.0.0) and searched all gene names, qualifier values, and keys that comprised the term “toxin.” To ensure that new candidate T/AT systems were not missed, we searched for VapBC and MazEF systems with BLASTP (E-value < 0.01, against previously identified PIN-like sequences belonging to potential toxin families) and HMMER (E-value < 0.01, against PF04014 and PF02452 models), respectively [77].

### Identification of RM systems in *S. lugdunensis*

Several tools have been developed to identify such coding sequences, the most complete being the REBASE database that provides an extensive in silico analysis of several bacterial genomes available in GenBank and allows identification and localization of RM systems [23]. Methylase specificity, nucleotide specificity, and closest neighbors were analyzed for each *S. lugdunensis* RM system.

## Additional files

Additional file 1: Pan-genome and core genome development projections for *S. lugdunensis* (A)*, S. epidermidis* (B)*,* and *S. aureus* (C). (DOCX 19 kb)

Additional file 2: ANI and average AAI of *S. lugdunensis* (A-B), *S. aureus* (C-D), and *S. epidermidis* (E-F) using the EDGAR interface. ANIs were calculated as the mean identity of all BLASTN matches that showed more than 30% overall sequence identity over at least 70% of an alignable region. (PPTX 667 kb)

Additional file 3: CRISPRs identified in 15 *S. lugdunensis* genomes using CRISPRFinder (available at: http://crispr.i2bc.paris-saclay.fr/). (DOCX 22 kb)

Additional file 4: Type IIIA CRISPR/Cas system alignments from *S. lugdunensis* strains HKU0901, N920143, VISLISI_27, VISLISI_33, and VISLISI_37. Nucleotide BLAST alignments were performed using Easyfig (v.2.2.2). (PDF 225 kb)

Additional file 5: MazEF operon comparison in 15 *S. lugdunensis* genomes, and in five other *Staphylococcus* species. All nucleotide BLAST comparisons were performed using Easyfig (v.2.2.2). (PDF 398 kb)

Additional file 6: Genomic coordinates of RM systems identified in 15 *S. lugdunensis* strains according to the REBASE database. (DOCX 16 kb)

Additional file 7: Pairwise alignment results according to EMBOSS Needle for the 3 loci of the Type I RM systems identified in *S. lugdunensis* VISLISI_33 and *S. aureus* MW2. (DOCX 574 kb)

Additional file 8: Genome accession numbers from GenBank database of staphylococci. Clinical and geographical origins of *S. lugdunensis* strains. (DOCX 18 kb)

## Abbreviations

AAI: Average amino acid identity
ANI: Average nucleotide identity
COG: Clusters of orthologous groups
CoNS: Coagulase-negative *Staphylococci*
CRISPR: Clustered regularly interspaced short palindromic repeats
DR: Direct repeat
HGT: Horizontal gene transfer
MGE: Mobile genetic element
MLST: Multilocus sequence typing
PRCI: Phage-related chromosomal island
RM: Restriction-modification
SaPI: *Staphylococcus aureus* pathogenicity island
SCC: Staphylococcal cassette chromosome
SRV: Score ratio values
T/AT: Toxin/antitoxin
