# Supplementary material for: Comparative genomic analysis of Staphylococcus lugdunensis shows a closed pan-genome and multiple barriers to horizontal gene transfer
Source: BMC Genomics. 2018 Aug 20;19:621. doi: 10.1186/s12864-018-4978-1 (PMC6102843; doi:10.1186/s12864-018-4978-1)
Supplement: Supplementary file 1 — Pan-genome and core genome development projections for S. lugdunensis (A), S. epidermidis (B), and S. aureus (C). (DOCX 19 kb) [file 12864_2018_4978_MOESM1_ESM.docx]

**Additional File 1.** Pan-genome and core genome development projections for *S. lugdunensis* (A)*, S. epidermidis* (B)*,* and S*. aureus* (C).

1. *S. lugdunensis*

| Pan-genome development extrapolations | | Core genome development extrapolations | |
| --- | --- | --- | --- |
| **Contigs** | **Mean number of genes** | **Contigs** | **Mean number of genes** |
| **1** | 2350.867 | **1** | 2350.733 |
| **2** | 2457.790 | **2** | 2243.943 |
| **3** | 2519.079 | **3** | 2197.218 |
| **4** | 2564.958 | **4** | 2166.502 |
| **5** | 2598.912 | **5** | 2144.259 |
| **6** | 2629.282 | **6** | 2123.687 |
| **7** | 2658.600 | **7** | 2106.083 |
| **8** | 2681.622 | **8** | 2091.204 |
| **9** | 2701.274 | **9** | 2075.306 |
| **10** | 2723.746 | **10** | 2061.202 |
| **11** | 2740.514 | **11** | 2047.187 |
| **12** | 2759.620 | **12** | 2033.501 |
| **13** | 2776.771 | **13** | 2020.562 |
| **14** | 2793.200 | **14** | 2008.067 |
| **15** | 2809.000 | **15** | 1996.000 |
| **Heap’s law extrapolation** | | **Heap’s law extrapolation** | |
| Growth exponent y:  0.066 (alpha 0.934) | | Converges to:  1944.613 | |
| Confidence interval (95%) for y:  0.065 to 0.067 | | Confidence interval (95%):  from 1932.995 to 1956.231 | |
| Fitted model:  2341.272 * x^0.066 | | Fitted model:  351.827 * exp(-x/8.956) + 1944.613 | |
| Lower confidence interval formula:  2336.425 * x^0.065 | | Lower confidence interval formula:  344.595 * exp(-x/8.303) + 1932.995 | |
| Upper confidence interval formula:  2346.119 * x^0.067 | | Upper confidence interval formula:   - 1. exp(-x/9.609) + 1956.231 | |

1. *S. epidermidis*

| Pan-genome development extrapolations | | Core genome development extrapolations | |
| --- | --- | --- | --- |
| **Contigs** | **Mean number of genes** | **Contigs** | **Mean number of genes** |
| **1** | 2326.231 | **1** | 2325.846 |
| **2** | 2602.564 | **2** | 2049.859 |
| **3** | 2807.664 | **3** | 1976.066 |
| **4** | 2975.326 | **4** | 1939.099 |
| **5** | 3124.906 | **5** | 1913.582 |
| **6** | 3259.246 | **6** | 1893.522 |
| **7** | 3362.444 | **7** | 1876.498 |
| **8** | 3463.782 | **8** | 1862.229 |
| **9** | 3561.474 | **9** | 1848.986 |
| **10** | 3642.098 | **10** | 1836.619 |
| **11** | 3720.487 | **11** | 1825.077 |
| **12** | 3794.077 | **12** | 1814.231 |
| **13** | 3864.000 | **13** | 1804.000 |
| **Heap’s Law projection** | | **Heap’s Law projection** | |
| Growth exponent y:  0.217 (alpha 0.783) | | Converges to:  1811.648 | |
| Confidence interval (95%) for y:  From 0.214 to 0.220 | | Confidence interval (95%):  from 1806.717 to 1816.578 | |
| Fitted model:  2208.227 * x^0.217 | | Fitted model:  355.405 * exp(-x/4.026) + 1811.648 | |
| Lower confidence interval formula:  2195.093 * x^0.214 | | Lower confidence interval formula:  346.024 * exp(-x/3.792) + 1806.717 | |
| Upper confidence interval formula:  2221.362 * x^0.220 | | Upper confidence interval formula:  364.786 * exp(-x/4.261) + 1816.578 | |

1. *S. aureus*

| Pan-genome development extrapolations | | Core genome development extrapolations | |
| --- | --- | --- | --- |
| **Contigs** | **Mean number genes** | **Contigs** | **Mean number of genes** |
| **1** | 2746.200 | **1** | 2745.600 |
| **2** | 2991.181 | **2** | 2501.190 |
| **3** | 3141.068 | **3** | 2401.745 |
| **4** | 3257.816 | **4** | 2341.805 |
| **5** | 3343.972 | **5** | 2301.360 |
| **6** | 3421.810 | **6** | 2267.926 |
| **7** | 3480.528 | **7** | 2245.045 |
| **8** | 3536.188 | **8** | 2217.903 |
| **9** | 3594.890 | **9** | 2200.338 |
| **10** | 3642.032 | **10** | 2179.493 |
| **11** | 3687.474 | **11** | 2161.890 |
| **12** | 3724.523 | **12** | 2145.620 |
| **13** | 3761.476 | **13** | 2129.448 |
| **14** | 3795.867 | **14** | 2113.933 |
| **15** | 3828.000 | **15** | 2099.000 |
| **Heap’s Law projection** | | **Heap’s Law projection** | |
| Growth exponent y:  0.123 (alpha 0.877) | | Converges to:  2098.510 | |
| Confidence interval (95%) for y:  from 0.121 to 0.124 | | Confidence interval (95%):  from 2091.072 to 2105.948 | |
| Fitted model:  2745.645 * x^0.123 | | Fitted model:  538.018 * exp(-x/5.228) + 2098.510 | |
| Lower confidence interval formula:  2739.282 * x^0.121 | | Lower confidence interval formula:  529.389 * exp(-x/4.964) + 2091.072 | |
| Upper confidence interval formula:  2752.008 * x^0.124 | | Upper confidence interval formula:  546.648 * exp(-x/5.491) + 2105.948 | |
