# Supplementary material for: Comparative genomic analysis of Staphylococcus lugdunensis shows a closed pan-genome and multiple barriers to horizontal gene transfer
Source: BMC Genomics. 2018 Aug 20;19:621. doi: 10.1186/s12864-018-4978-1 (PMC6102843; doi:10.1186/s12864-018-4978-1)
Supplement: Supplementary file 2 — ANI and average AAI of S. lugdunensis (A-B), S. aureus (C-D), and S. epidermidis (E-F) using the EDGAR interface. ANIs were calculated as the mean identity of all BLASTN matches that showed more than 30% overall sequence identity over at least 70% of an alignable region. (PPTX 667 kb) [file 12864_2018_4978_MOESM2_ESM.pptx]

## Slide 1
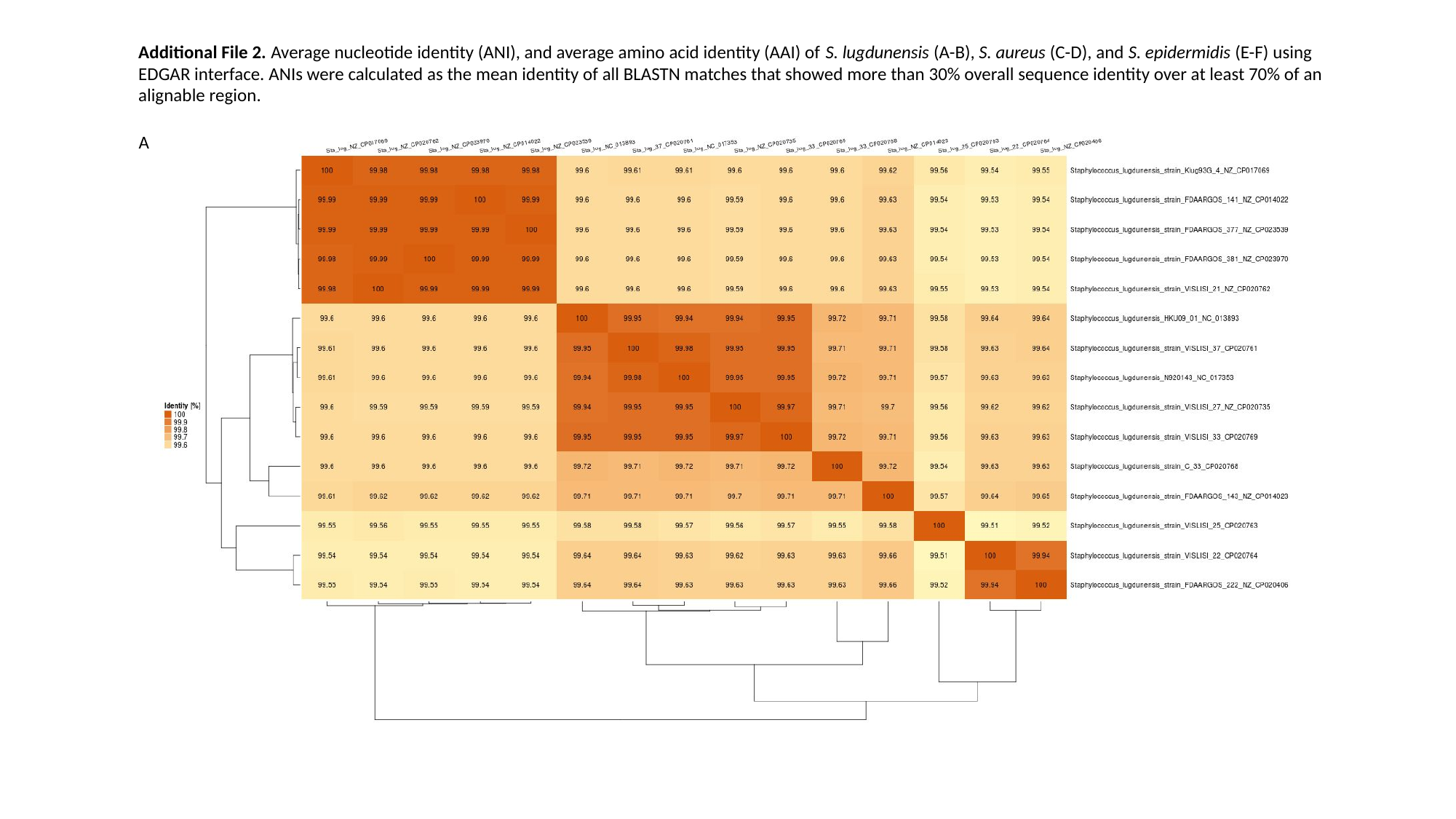

Additional File 2. Average nucleotide identity (ANI), and average amino acid identity (AAI) of S. lugdunensis (A-B), S. aureus (C-D), and S. epidermidis (E-F) using EDGAR interface. ANIs were calculated as the mean identity of all BLASTN matches that showed more than 30% overall sequence identity over at least 70% of an alignable region.
A

## Slide 2
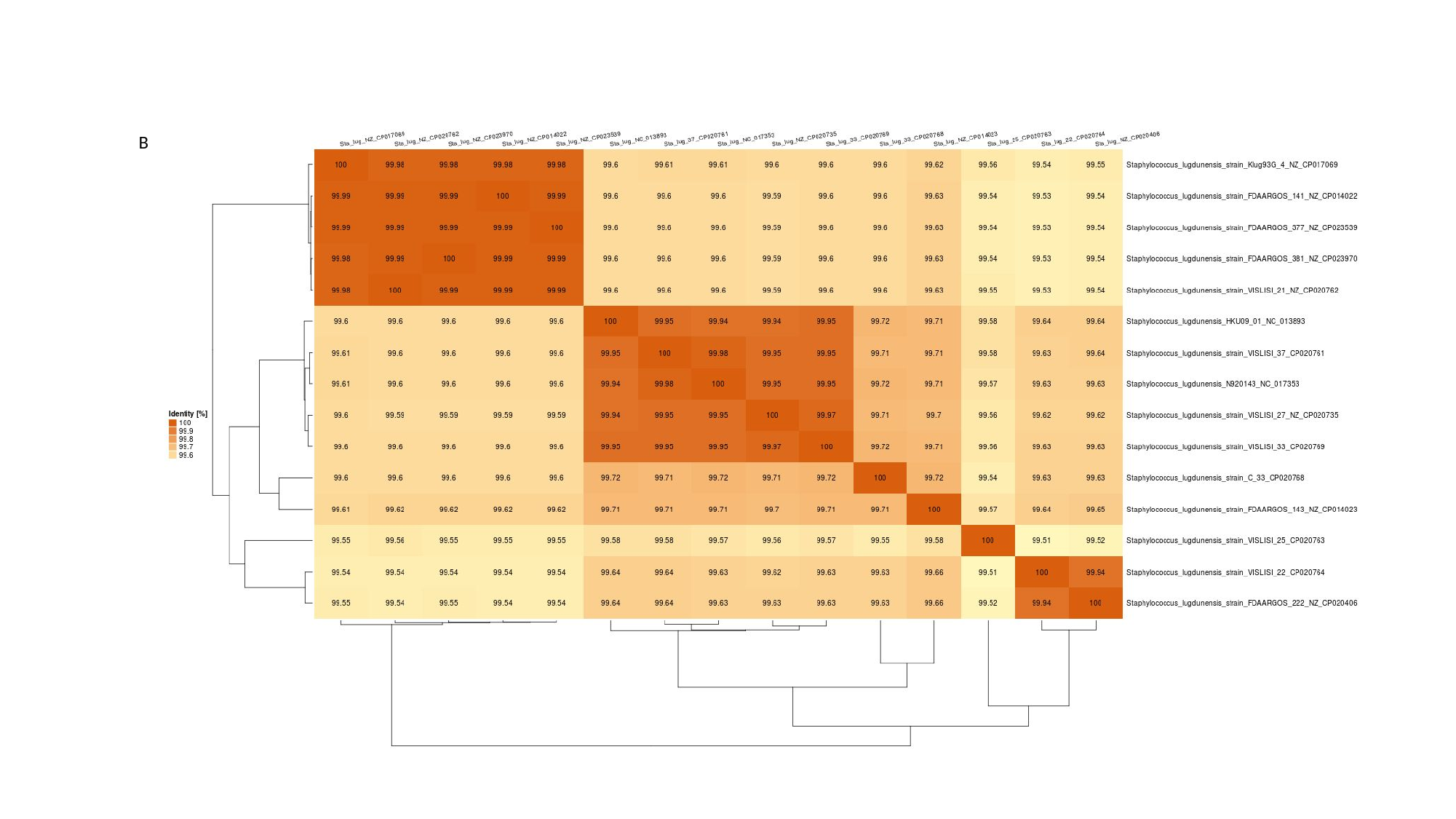

B

## Slide 3
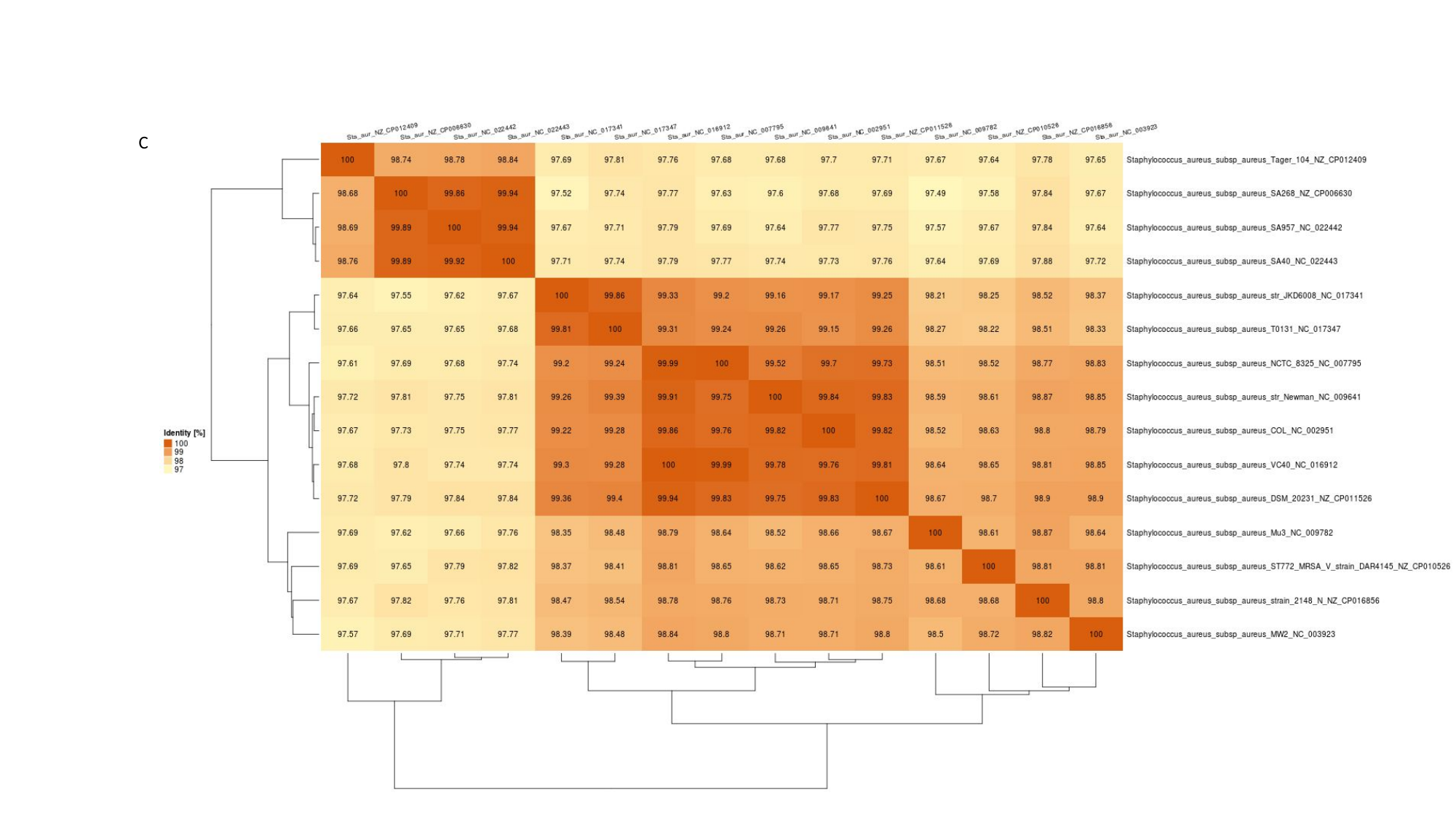

C

## Slide 4
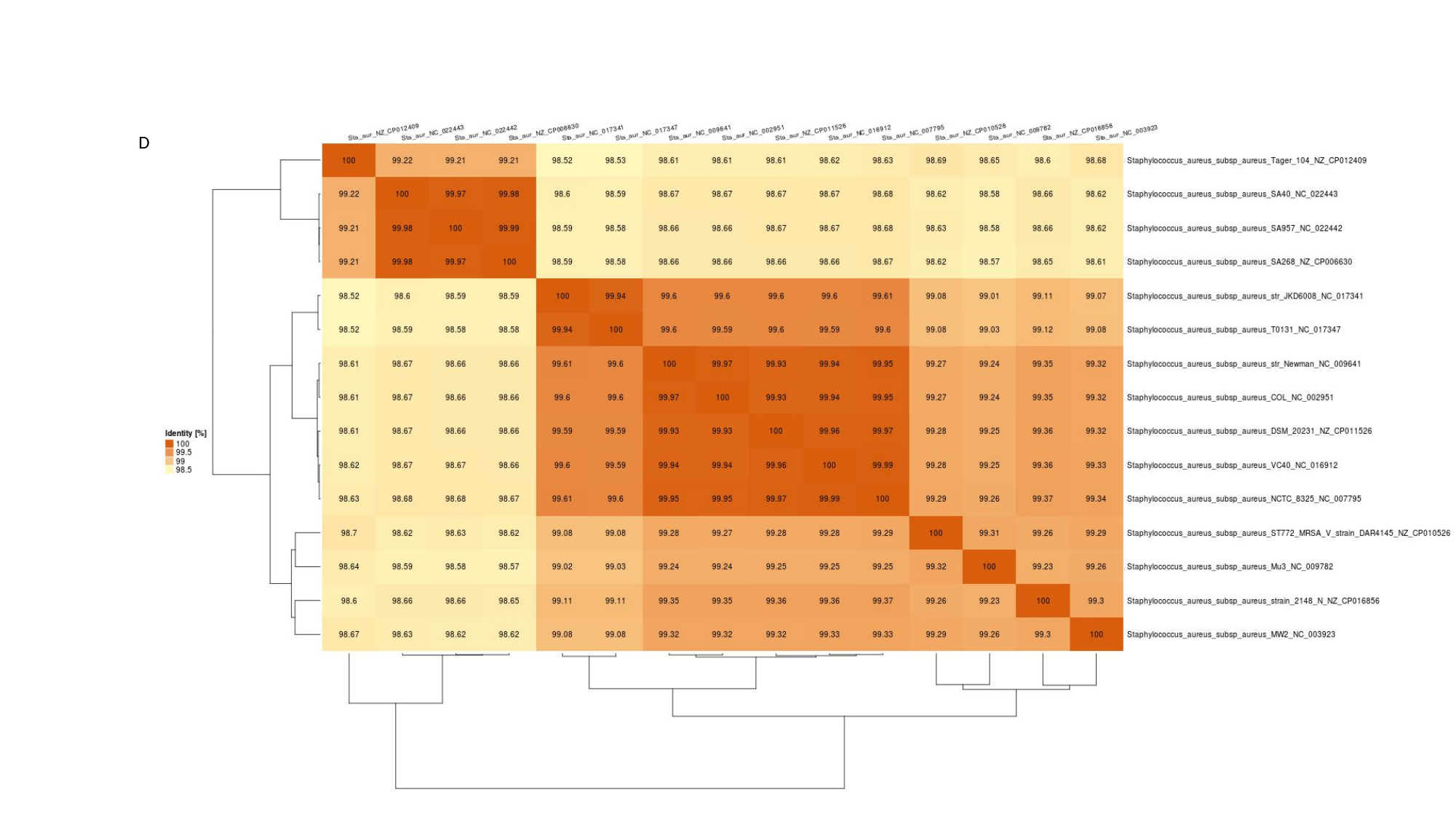

D

## Slide 5
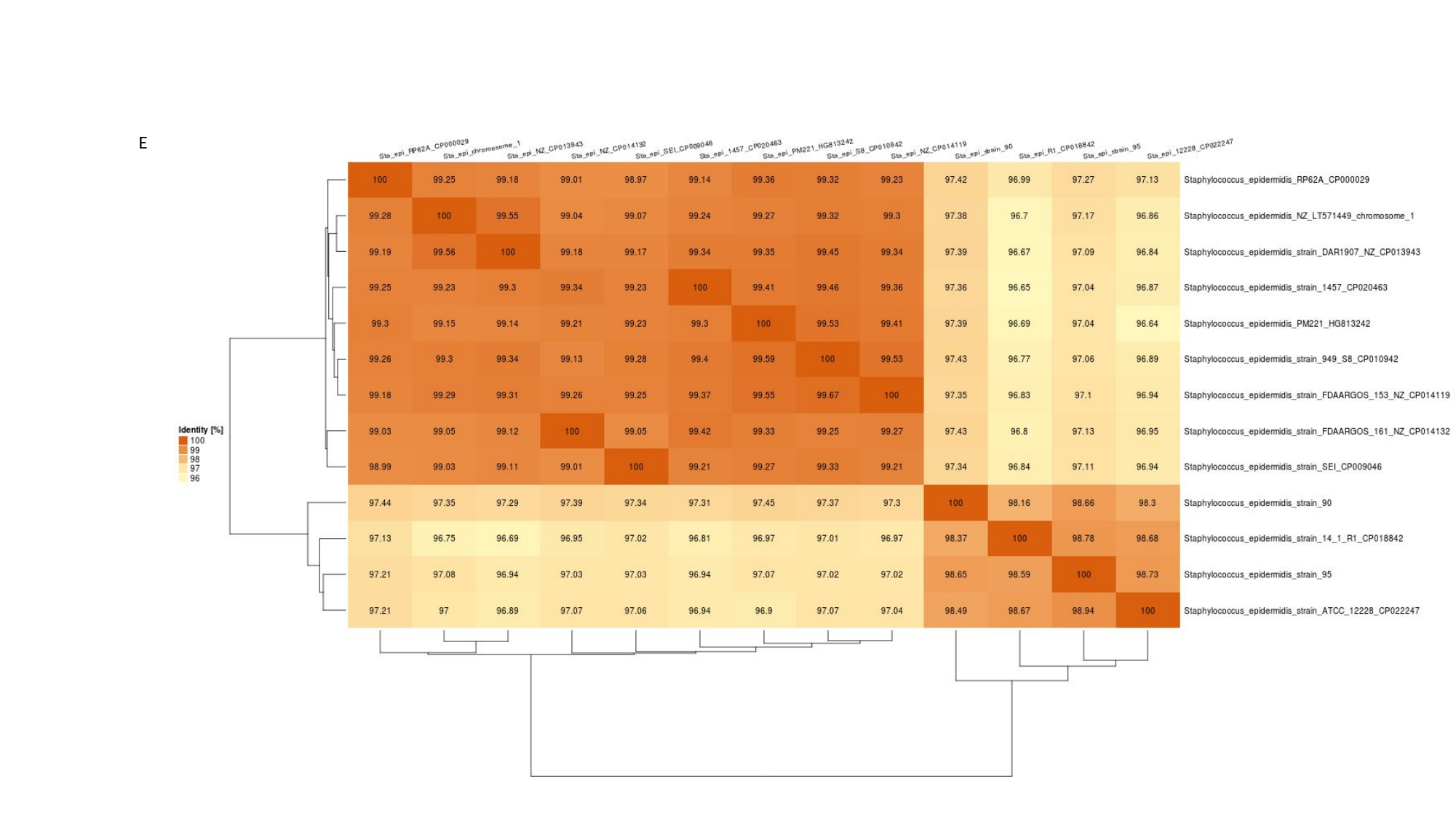

E

## Slide 6
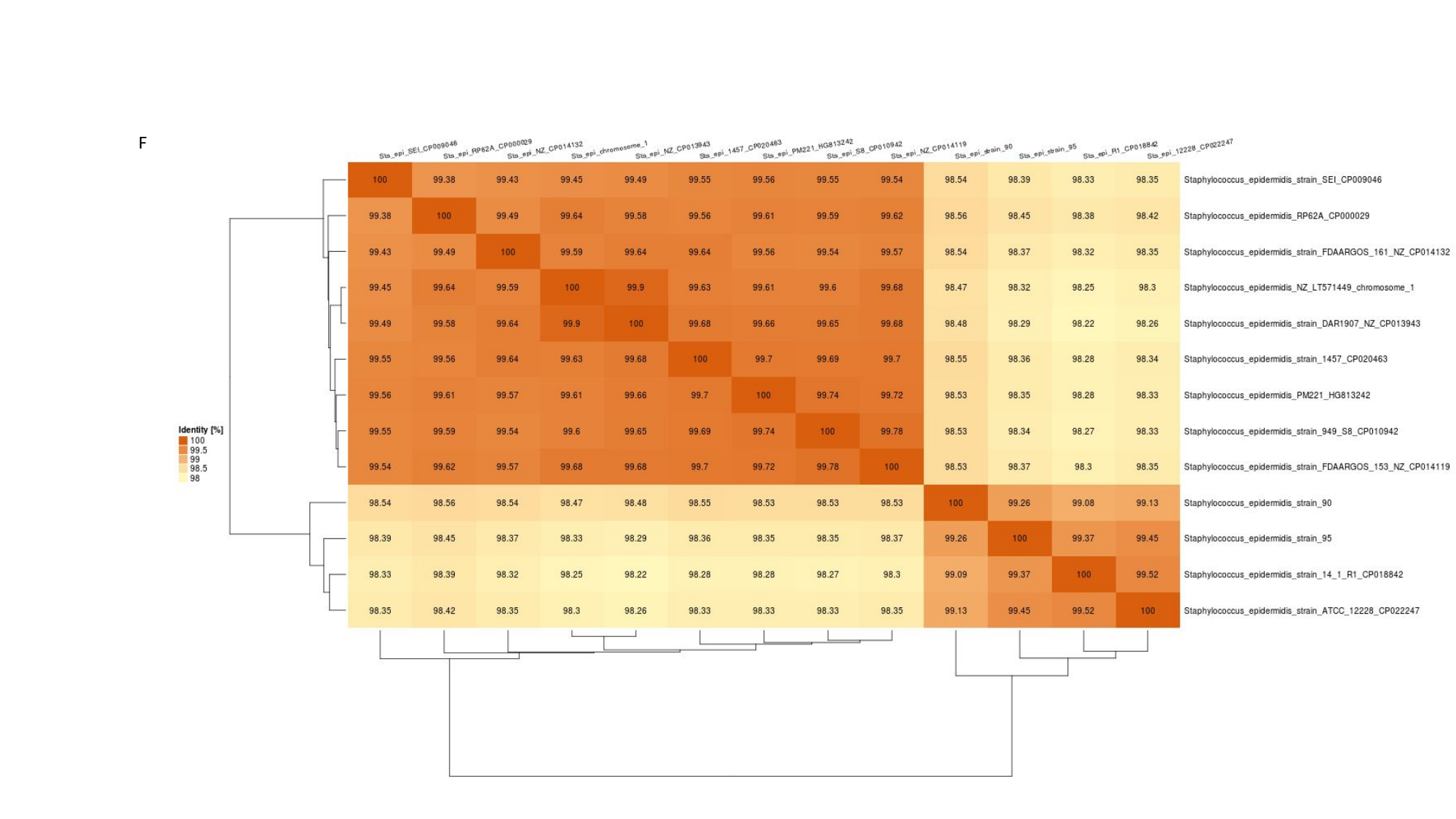

F
