# Supplementary material for: Comparative genomic analysis of Staphylococcus lugdunensis shows a closed pan-genome and multiple barriers to horizontal gene transfer
Source: BMC Genomics. 2018 Aug 20;19:621. doi: 10.1186/s12864-018-4978-1 (PMC6102843; doi:10.1186/s12864-018-4978-1)
Supplement: Supplementary file 3 — CRISPRs identified in 15 S. lugdunensis genomes using CRISPRFinder (available at: http://crispr.i2bc.paris-saclay.fr/). (DOCX 22 kb) [file 12864_2018_4978_MOESM3_ESM.docx]

**Additional File 3.** CRISPRs identified in 15 *S. lugdunensis* genomes using CRISPERFinder (available at: <http://crispr.i2bc.paris-saclay.fr/)>

| Strain | Number of confirmed CRISPRs | Number of questionable CRISPRs |
| --- | --- | --- |
| HKU0901 | 1 | 9 |
| N920143 | 1 | 9 |
| FDAARGOS_141 | 0 | 4 |
| FDAARGOS_143 | 0 | 1 |
| FDAARGOS_222 | 0 | 4 |
| Klug93G-4 | 0 | 5 |
| FDAARGOS_377 | 0 | 5 |
| FDAARGOS_381 | 0 | 5 |
| VISLISI_21 | 0 | 5 |
| VISLISI_22 | 0 | 4 |
| VISLISI_25 | 0 | 5 |
| VISLISI_27 | 1 | 11 |
| VISLISI_33 | 1 | 11 |
| VISLISI_37 | 1 | 9 |
| C33 | 1 | 4 |

Confirmed CRISPRS characteristics

**HKU0901**

CRISPR start position: 29464

CRISPR end position: 29929

DR consensus: AATCAGAGAATACCCCGTATAAAAGGGGACGAGAAC

DR length: 36

Number of spacers: 6

| Spacer begin position | Spacer length | Spacer sequence | Spacer ID^1^ |
| --- | --- | --- | --- |
| 29500 | 37 | TTCTGTATTTCTATTAACCAATTCATAAGTGTCATTA | S1 |
| 29573 | 35 | TTCATCATATCCTTTTATATAGTTTTTGTTTTGTT | S2 |
| 29644 | 35 | CCAGTTGCTATTTTATTTGTCAACCATTTTATTAA | S3 |
| 29715 | 32 | TAGAATGTTTAAACAAGGTGTTTCAAAACCTG | S4 |
| 29783 | 39 | AACATTTTATTTACGTCTGTTTTTGCCTCTACTAAGTAA | S5 |
| 29858 | 36 | TCGCTATTCAATAAGTTTTGTATTTGTTGTCTCATT | S6 |

1 Identification number

**N920143**

CRISPR start position: 63455

CRSPR end position: 63777

DR consensus: AATCAGAGAATACCCCGTATAAAAGGGGACGAGAAC

DR length: 36

Number of spacers: 4

| Spacer begin position | Spacer length | Spacer sequence | Spacer ID |
| --- | --- | --- | --- |
| 63491 | 35 | TAACACCTTTGATTTTATATCTTTTTCCATCAAAT | S7 |
| 63562 | 37 | CCAAATAATACTATTAATTTATCTGTTAATGCTGTGG | S8 |
| 63635 | 35 | TGGTAAATCTTCTACACTGTCGACTGGGTCTTCTA | S9 |
| 63706 | 36 | TGTTCCAATTAGCTAAAGATGTTATGGACGGCAAGA | S10 |

**VISLISI_27**

CRISPR start position: 20682

CRISPR end position: 21217

DR consensus: AATCAGAGAATACCCCGTATAAAAGGGGACGAGAACT

DR length: 37

Number of spacers: 7

| Spacer begin position | Spacer length | Spacer sequence | Spacer ID |
| --- | --- | --- | --- |
| 20719 | 34 | ATCAAAACATCATTTTTAATATATTTTAATTGTT | S11 |
| 20790 | 34 | GAATTTCACTTGCGAATTTTAAATAACTATCCCC | S12 |
| 20861 | 33 | TTATTAGCTATTTCATTATCTATATTATCACTA | S13 |
| 20931 | 36 | TCTGTATTTCTATTAACCAATTCATAAGTGTCATTA | S1 |
| 21004 | 34 | TCATCATATCCTTTTATATAGTTTTTGTTTTGTT | S2 |
| 21075 | 34 | GGTAAATCTTCTACACTGTCGACTGGGTCTTCTA | S9 |
| 21146 | 35 | CGCTATTCAATAAGTTTTGTATTTGTTGTCTCATT | S6 |

**VISLISI_33**

CRISPR start position: 20195

CRISPR end position: 20952

DR consensus: AATCAGAGAATACCCCGTATAAAAGGGGACGAGAAC

DR length: 36

Number of spacers: 10

| Spacer begin position | Spacer length | Spacer sequence | Spacer ID |
| --- | --- | --- | --- |
| 20231 | 40 | CCACCTTTACTTTTCGTCATATCATTTTTCTTTTGAGCGA | S14 |
| 20307 | 36 | TCCCAGTTTGGTAAGTTCATTTTTAATCATCCTTTT | S15 |
| 20379 | 36 | ATAATCAATTGTTAAATACATTTCATTTGGATTAAT | S16 |
| 20451 | 37 | TTCTGTATTTCTATTAACCAATTCATAAGTGTCATTA | S1 |
| 20524 | 35 | TTCATCATATCCTTTTATATAGTTTTTGTTTTGTT | S2 |
| 20595 | 35 | TGGTAAATCTTCTACACTGTCGACTGGGTCTTCTA | S9 |
| 20666 | 35 | TGTGCACTTTTACACTATTAGTTTAAATATCTCCA | S17 |
| 20737 | 37 | CTTTATATACAGTAGTTAATGACTTATGAAAGGTTTC | S18 |
| 20810 | 35 | CCAGTTGCTATTTTATTTGTCAACCATTTTATTAA | S3 |
| 20881 | 36 | TCGCTATTCAATAAGTTTTGTATTTGTTGTCTCATT | S6 |

**VISLISI_37**

CRISPR start position: 182355

CRISPR end position: 182608

DR consensus: CTAATCAGAGAATACCCCGTATAAAAGGGGACGAGAAC

DR length: 38

Number of spacers: 3

| Spacer begin position | Spacer length | Spacer sequence | Spacer ID |
| --- | --- | --- | --- |
| 182393 | 35 | ATATTTTACTATATGCTCATATCCGCCGTTTGTTT | S18 |
| 182466 | 33 | TGGTAAATCTTCTACACTGTCGACTGGGTCTTC | S9 |
| 182537 | 34 | TGTTCCAATTAGCTAAAGATGTTATGGACGGCAA | S10 |

**C33**

CRISPR start position: 98485

CRISPR end position: 99241

DR consensus: GTTTTAGTACTCTGTAATTTTAGGTATAAGTGATAC

DR length: 36

Number of spacers: 11

| Spacer begin position | Spacer length | Spacer sequence |
| --- | --- | --- |
| 98521 | 29 | TGTTCGTCCATTTGTCTTTAAAATTATAT |
| 98586 | 30 | TTGGTGGCGGAGCTATCATTGCAGTAGCAG |
| 98652 | 29 | GTATAAAAAAACACCGTATCAACTAAGAT |
| 98717 | 30 | ATTATGACATCAATAGTATCACTTTCCCAG |
| 98783 | 29 | TGAAGGTAAAGACAGAGGACATAGTCCTA |
| 98848 | 29 | CGGTTTAGGTGCTGTTGCAATGGCTGGAA |
| 98913 | 30 | GTTTTTCGAATGAGTTGATAGTGACGTCTG |
| 98979 | 30 | TCACATCATTCGAACGTAAGAATGAGGCTG |
| 99045 | 29 | TCTGTGTTCAATCCATGCAGTCTGTCCAA |
| 99110 | 30 | GTATTGAAGATGTAAGAAAAGACACGGTTC |
| 99176 | 30 | ACTAAAAACTGACTGCTACGGCAGAGATCA |

Questionable CRISPRS

Recurrent motif

DR: TGATTGAGAAGTCAGTCGAAAAAC

DR length: 24

Spacer sequence

TATTGCAAAAGAAAAACGATTATGTATAATAAAGTTATAAAT

Spacer length: 42
