# Supplementary material for: Comparative genomic analysis of Staphylococcus lugdunensis shows a closed pan-genome and multiple barriers to horizontal gene transfer
Source: BMC Genomics. 2018 Aug 20;19:621. doi: 10.1186/s12864-018-4978-1 (PMC6102843; doi:10.1186/s12864-018-4978-1)

Additional file 6. Type IIIA CRISPR/Cas system alignments from *S. lugdunensis* strains HKU0901, N920143, VISLISI_27, VISLISI_33, and VISLISI_37. Nucleotide BLAST alignments were performed using Easyfig (v.2.2.2).

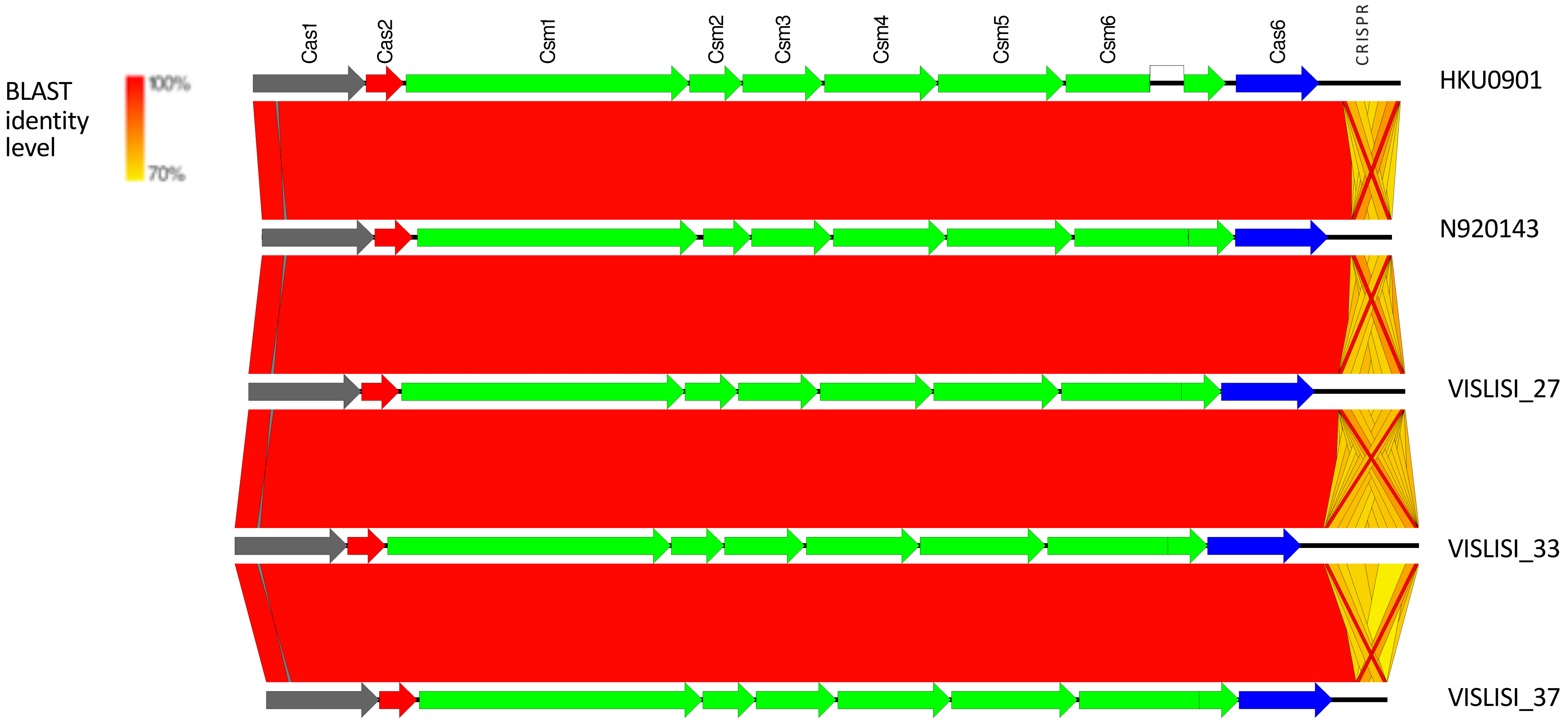

Supplement: Supplementary file 4 — Type IIIA CRISPR/Cas system alignments from S. lugdunensis strains HKU0901, N920143, VISLISI_27, VISLISI_33, and VISLISI_37. Nucleotide BLAST alignments were performed using Easyfig (v.2.2.2). (PDF 225 kb) [file 12864_2018_4978_MOESM4_ESM.pdf]
