# Supplementary material for: Comparative genomic analysis of Staphylococcus lugdunensis shows a closed pan-genome and multiple barriers to horizontal gene transfer
Source: BMC Genomics. 2018 Aug 20;19:621. doi: 10.1186/s12864-018-4978-1 (PMC6102843; doi:10.1186/s12864-018-4978-1)
Supplement: Supplementary file 5 — MazEF operon comparison in 15 S. lugdunensis genomes, and in five other Staphylococcus species. All nucleotide BLAST comparisons were performed using Easyfig (v.2.2.2). (PDF 398 kb) [file 12864_2018_4978_MOESM5_ESM.pdf]

Additional file 7. MazEF operon comparison in 15 *S. lugdunensis* genomes, and in five other *Staphylococcus* species. All nucleotide BLAST comparisons were performed using Easyfig (v.2.2.2)

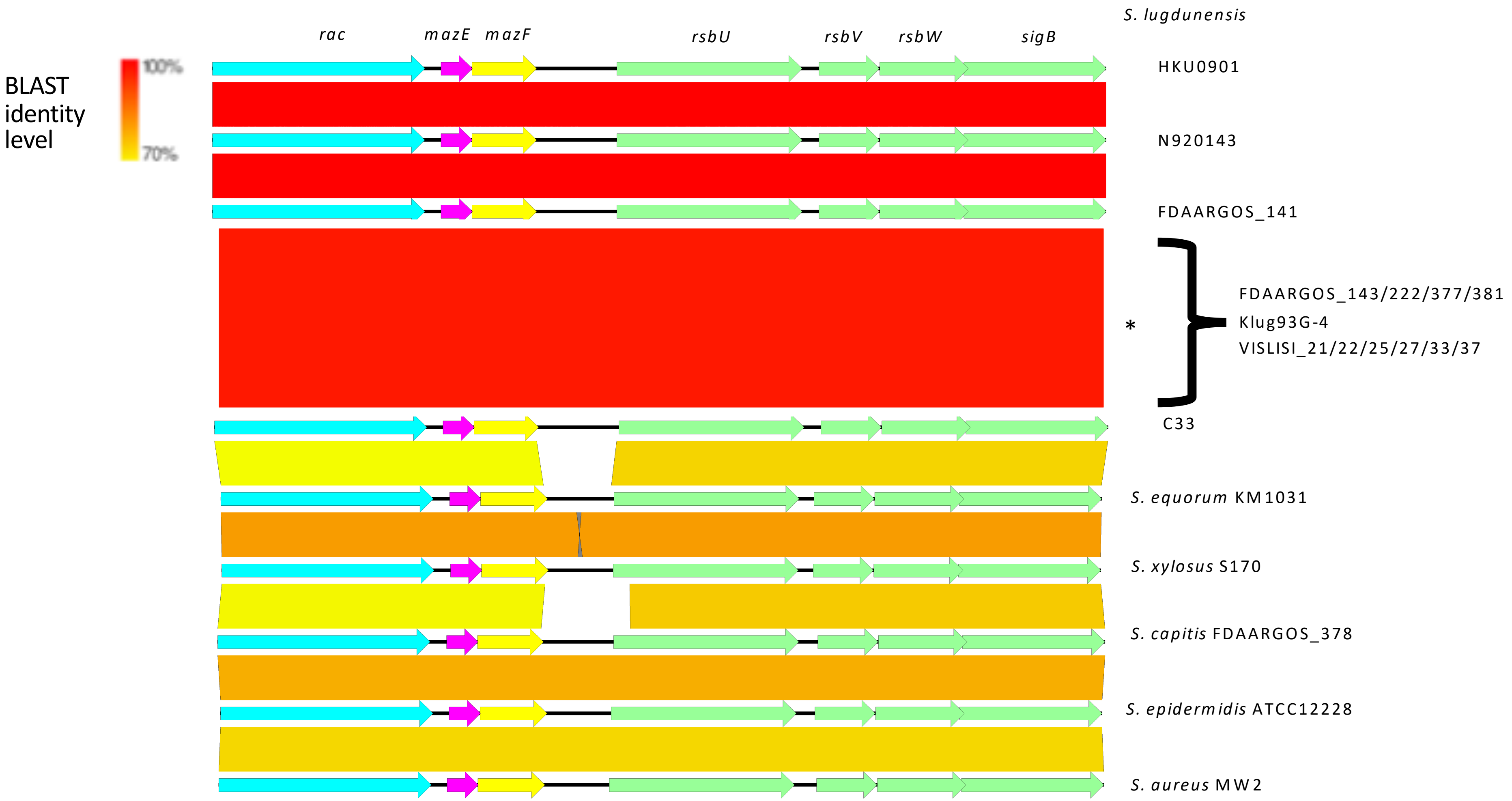

* Image was modified for better visualisation. All *S. lugdunensis* strains had the same BLAST identity level.
