# Supplementary material for: Comparative genomic analysis of Staphylococcus lugdunensis shows a closed pan-genome and multiple barriers to horizontal gene transfer
Source: BMC Genomics. 2018 Aug 20;19:621. doi: 10.1186/s12864-018-4978-1 (PMC6102843; doi:10.1186/s12864-018-4978-1)
Supplement: Supplementary file 6 — Genomic coordinates of RM systems identified in 15 S. lugdunensis strains according to the REBASE database. (DOCX 16 kb) [file 12864_2018_4978_MOESM6_ESM.docx]

**Additional File 4.** Genomic coordinates of Restriction-Modification systems identified in 15 *S. lugdunensis* strains according to REBASE database.

***S. lugdunensis* HKU0901**

| **Type** | **Gene** | **Name** | **Coordinates** |
| --- | --- | --- | --- |
| I | S | S.SluHKORF654P | 715359-716534 c |
| I | M | M.SluHKORF654P | 716527-718083 c |
| I | R | SluHKORF654P | 718182-720968 c |

***S. lugdunensis* N902143**

| **Type** | **Gene** | **Name** | **Coordinates** |
| --- | --- | --- | --- |
| I | S | S.Slu920143ORF6520P | 710881-712056 c |
| I | M | M.Slu920143ORF6520P | 712049-713605 c |
| I | R | Slu920143ORF6520P | 713704-716490 c |

***S. lugdunensis* FDAARGOS_143**

| **Type** | **Gene** | **Name** | **Coordinates** |
| --- | --- | --- | --- |
| I | R | Slu143ORF6170P | 1261986-1264772 |
| I | M | M.Slu143ORF6170P | 1264871-1266427 |
| I | S | S.Slu143ORF6170P | 1266420-1267604 |
|  |  |  |  |
| I | R | Slu143ORF9045P | 1935169-1938288 c |
| I | S | S.Slu143ORF9045P | 1938272-1939567 c |
| I | M | M.Slu143ORF9045P | 1939557-1941071 c |

***S. lugdunensis* FDAARGOS_222**

| **Type** | **Gene** | **Name** | **Coordinates** |
| --- | --- | --- | --- |
| II | R | Slu222ORF1035P | 242626-244095 |
| II | M | M.Slu222ORF1035P | 244171-245409 |

***S. lugdunensis* VISLISI_22**

| **Type** | **Gene** | **Name** | **Coordinates** |
| --- | --- | --- | --- |
| II | R | Slu22ORF3035P | 678794-680263 |
| II | M | M.Slu22ORF3035P | 680339-681577 |

***S. lugdunensis* VISLIS_25**

| **Type** | **Gene** | **Name** | **Coordinates** |
| --- | --- | --- | --- |
| II | M | M1.Slu25ORF2125P | 481110-482117 c |
| II | M | M2.Slu25ORF2125P | 482092-483105 c |
| II | R | Slu25ORF2125P | 483229-484431 |

***S. lugdunensis* VISLISI_27**

| **Type** | **Gene** | **Name** | **Coordinates** |
| --- | --- | --- | --- |
| I | S | S.Slu27ORF3020P | 672411-673586 c |
| I | M | M.Slu27ORF3020P | 673579-675135 c |
| I | R | Slu27ORF3020P | 675234-678020 c |
|  |  |  |  |
| I | R | Slu27ORF12355P | 2562443-2565472 c |
| I | S | S.Slu27ORF12355P | 2565486-2566748 c |
| I | M | M.Slu27ORF12355P | 2566753-2568540 c |

***S. lugdunensis* VISLISI_33**

| **Type** | **Gene** | **Name** | **Coordinates** |
| --- | --- | --- | --- |
| I | S | S.Slu33ORF3050P | 670534-671709 c |
| I | M | M.Slu33ORF3050P | 671702-673258 c |
| I | R | Slu33ORF3050P | 673357-676143 c |

***S. lugdunensis* C_33 pVISLISI_5**

| **Type** | **Gene** | **Name** | **Coordinates** |
| --- | --- | --- | --- |
| II | M | M.SluC33ORF60P | 9827-10576 |
| II | R | SluC33ORF65P | 10557-12089 |
