# Supplementary material for: Comparative genomic analysis of Staphylococcus lugdunensis shows a closed pan-genome and multiple barriers to horizontal gene transfer
Source: BMC Genomics. 2018 Aug 20;19:621. doi: 10.1186/s12864-018-4978-1 (PMC6102843; doi:10.1186/s12864-018-4978-1)

**Additional File 5.** Pairwise alignment results according to EMBOSS Needle for the three loci of the Type I Restriction-Modification systems identified in *S. lugdunensis* VISLISI_33, and *S. aureus* MW2.

Locus *hsdM*


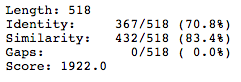


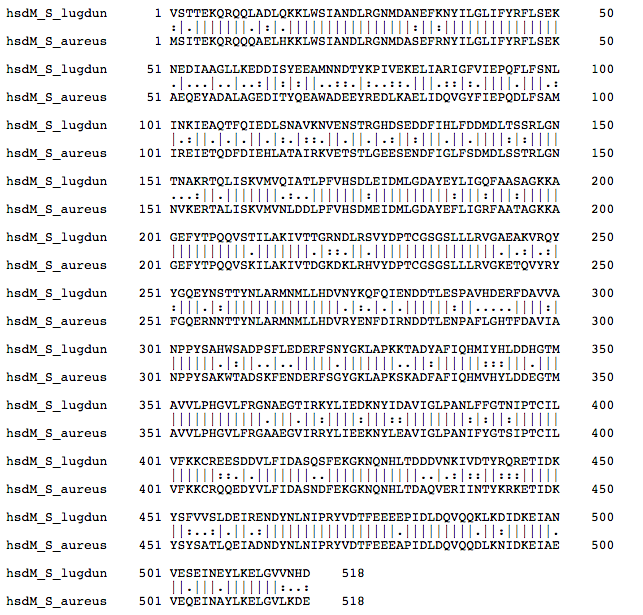


Locus *hsdR*, The green box indicates the motif essential for DNA cleavage, and the red boxes indicate the motifs essential for the DNA translocation (according to Roberts et al. 2013).


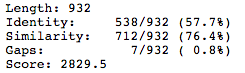

Locus *hsdS*


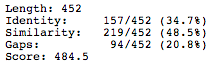


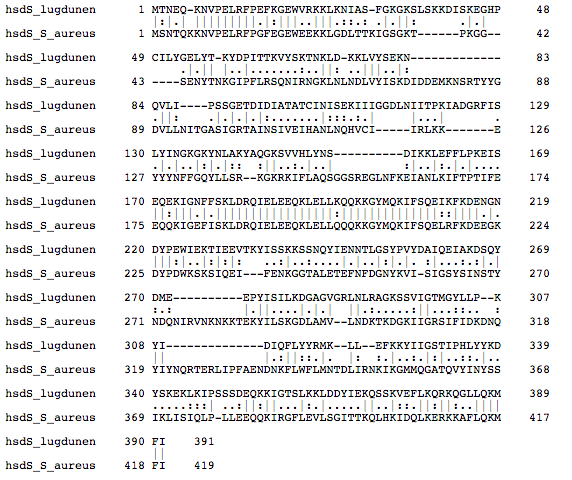

Supplement: Supplementary file 7 — Pairwise alignment results according to EMBOSS Needle for the 3 loci of the Type I RM systems identified in S. lugdunensis VISLISI_33 and S. aureus MW2. (DOCX 574 kb) [file 12864_2018_4978_MOESM7_ESM.docx]
