# Supplementary material for: Comparative genomic analysis of Staphylococcus lugdunensis shows a closed pan-genome and multiple barriers to horizontal gene transfer
Source: BMC Genomics. 2018 Aug 20;19:621. doi: 10.1186/s12864-018-4978-1 (PMC6102843; doi:10.1186/s12864-018-4978-1)
Supplement: Supplementary file 8 — Genome accession numbers from GenBank database of staphylococci. Clinical and geographical origins of S. lugdunensis strains. (DOCX 18 kb) [file 12864_2018_4978_MOESM8_ESM.docx]

**Additional File 8.** Genome accession numbers from GenBank database of staphylococci. Clinical and geographical origins of *S. lugdunensis* strains.

| *Staphylococcus* species | Strains | NCBI accession numbers | Isolation source | Country | Date of isolation |
| --- | --- | --- | --- | --- | --- |
| *S. lugdunensis* | HKU0901 | NC_013893 | Clinical specimen | Hong Kong | 2010 |
|  | N920143 | NC_017353 | Breast abscess | France | 2009 |
|  | FDAARGOS_141 | NZ_CP014022 | Right elbow swab | USA | 2014 |
|  | FDAARGOS_143 | NZ_CP014023 | Abscess | USA | 2014 |
|  | FDAARGOS_222 | NZ_CP020406 | Axillary lymph node | France | 1988 |
|  | Klug93G-4 | NZ_CP017069 | Groin swab | Hong Kong | 2014 |
|  | FDAARGOS_377 | NZ_CP023539 | Abscess | USA | 2015 |
|  | FDAARGOS_381 | NZ_CP023970 | Abscess | USA | 2015 |
|  | VISLISI_21 | NZ_CP020762 | Blood culture | France | 2015 |
|  | VISLISI_22 | NZ_CP020764 | Blood culture | France | 2015 |
|  | VISLISI_25 | NZ_CP020763 | Prosthesis sonication | France | 2015 |
|  | VISLISI_27 | NZ_CP020735 | Prosthesis sonication | France | 2014 |
|  | VISLISI_33 | NZ_CP020769 | Liver abscess | France | 2015 |
|  | VISLISI_37 | NZ_CP020761 | Blood culture | France | 2015 |
|  | C33 | NZ_CP020768 | Cutaneous swab | France | 2015 |

| *Staphylococcus* species | Strains | NCBI accession numbers |
| --- | --- | --- |
| *S. aureus* | DSM_20231 | NZ_CP011526 |
|  | MW2 | NC_003923 |
|  | Mu3 | NC_009782 |
|  | NCTC_832547 | NC_007795 |
|  | SA268 | NZ_CP006630 |
|  | SA40 | NC_022443 |
|  | SA957 | NC_022442 |
|  | ST772_MRSA | NZ_CP010526 |
|  | T0131 | NC_017347 |
|  | Tager_104 | NZ_CP012409 |
|  | VC40 | NC_016912 |
|  | JKD6008 | NC_017341 |
|  | Newman | NC_009641 |
|  | 2148 | NZ_CP016856 |
|  | Col | NC_002951.2 |
|  |  |  |
| *S. epidermidis* | ATCC12228 | NZ_CP022247 |
|  | RP62A | NC_002976 |
|  | PM221 | NZ_HG813242 |
|  | SEI | NZ_CP009046 |
|  | 14.1.R1 | CP18842 |
|  | 1457 | NZ_CP020463 |
|  | SE90 | NZ_CP024408 |
|  | SE95 | NZ_CP024437 |
|  | DAR1907 | NZ_CP013943 |
|  | FDAARGOS_153 | NZ_CP014119 |
|  | FDAARGOS_161 | NZ_CP014132 |
|  | BPH0662 | NT_LT571449 |
|  | 949_S8 | CP010942 |
|  |  |  |
| *S. equorum* | KM1031 | NZ_CP013980.1 |
|  |  |  |
| *S. xylosus* | S170 | NZ_CP013922.1 |
|  |  |  |
| *S. capitis* | *FDAARGOS_378* | NZ_CP023966.1 |
|  |  |  |
| *S. haemolyticus* | JCSC1435 | NC_007168.1 |
